# Reversible electrical percolation in a stretchable and self-healable silver-gradient nanocomposite bilayer

**DOI:** 10.1038/s41467-022-32966-x

**Published:** 2022-09-05

**Authors:** Jinhong Park, Duhwan Seong, Yong Jun Park, Sang Hyeok Park, Hyunjin Jung, Yewon Kim, Hyoung Won Baac, Mikyung Shin, Seunghyun Lee, Minbaek Lee, Donghee Son

**Affiliations:** 1 The Institute for Basic Science, Inha University, Incheon, 22212 Republic of Korea; 2 Department of Physics, Inha University, Incheon, 22212 Republic of Korea; 3 Department of Electrical and Computer Engineering, Sungkyunkwan University, Suwon, 16419 Republic of Korea; 4 Center for Neuroscience Imaging Research, Institute for Basic Science (IBS), Suwon, 16419 Republic of Korea; 5 Department of Intelligent Precision Healthcare Convergence, Sungkyunkwan University, Suwon, 16419 Republic of Korea; 6 Department of Electronic Engineering, Kyunghee University, Yongin, 17104 Republic of Korea; 7 Department of Superintelligence Engineering, Sungkyunkwan University, Suwon, 16419 Republic of Korea

**Keywords:** Electronic devices, Electrical and electronic engineering

## Abstract

The reversibly stable formation and rupture processes of electrical percolative pathways in organic and inorganic insulating materials are essential prerequisites for operating non-volatile resistive memory devices. However, such resistive switching has not yet been reported for dynamically cross-linked polymers capable of intrinsic stretchability and self-healing. This is attributable to the uncontrollable interplay between the conducting filler and the polymer. Herein, we present the development of the self-healing, stretchable, and reconfigurable resistive random-access memory. The device was fabricated via the self-assembly of a silver-gradient nanocomposite bilayer which is capable of easily forming the metal-insulator-metal structure. To realize stable resistive switching in dynamic molecular networks, our device features the following properties: i) self-reconstruction of nanoscale conducting fillers in dynamic hydrogen bonding for self-healing and reconfiguration and ii) stronger interaction among the conducting fillers than with polymers for the formation of robust percolation paths. Based on these unique features, we successfully demonstrated stable data storage of cardiac signals, damage-reliable memory triggering system using a triboelectric energy-harvesting device, and touch sensing via pressure-induced resistive switching.

## Abstract (web summary)

Smart healthcare devices, which interacts with the human body by recording, analyzing and processing physiological signals, need soft and biocompatible electronics. Here, Son et al. report a self-healable and stretchable resistive switching random-access memory made of a Ag-gradient nanocomposite.

## Introduction

Closed-loop electronic healthcare systems have been explosively spotlighted owing to their interactive functions that enable them to record physiological signals from the human body, analyze these signals using big data processing, and even deliver feedback therapy to abnormal tissues^1–3^. More recently, neuromorphic engineering, with respect to the way in which personal data can be stably stored or precisely processed, has been suggested to be of key importance for the realization of such smart bioelectronic systems^4,5^. In this regard, a soft resistive switching random-access memory (RRAM) device with low power consumption, high switching speed, and long retention time is a promising candidate that can either store massive amounts of information or acts as a neuromorphic module with embedded artificial intelligence^3,6,7^. Furthermore, its 2-terminal structure is more efficient for achieving high-density storage cells than floating-gate/charge-trap memory devices. However, the development of soft, biocompatible, high-performance RRAM with a mechanical modulus comparable to that of various living tissues is an essential prerequisite for realizing tissue-like bioelectronic systems. To meet this requirement, many research groups have adopted either strain-dissipative structural designs or intrinsically stretchable materials^8–15^. Although these efforts have shown the feasibility of the strain-insensitive stable electrical operation of RRAM, the mechanical reliability of the conventional stretchable materials (ultrathin wavy polyimide or non-healable viscoelastic materials) acting as stretchable substrates would be vulnerable to long-term mechanical stresses originating from repetitive movement of various organs (e.g. skin, heart, tendon, and peripheral nerve).

This challenge can be overcome by applying soft stretchable polymeric materials with high toughness and self-healing properties to the electrodes and resistive switching materials of RRAM devices^16–18^. Although self-healing materials have various mechanisms to recover autonomously, self-healing polymers based on dynamic hydrogen bonding would be more beneficial for forming the underlying polymeric resistive switching materials of the RRAM than those using capsule-type or metal coordination methods^19,20^. This is because these self-healing polymers offer the skin-like modulus, the efficient dissipation of strain energy, low chemical reactivity, and even high biocompatibility^21,22^. Specifically, chemical stability at resistive switching interfaces is more important to precisely control the long-term electrical performance of the RRAM^23,24^. However, the dynamic behavior induced by the low glass transition temperature (at room temperature) may potentially intervene in reversibly forming conducting filamentary paths in the RRAM. Furthermore, the stable formation and breakdown of the electrical percolation pathways in RRAM should be maintained in various stretching modes. These critical challenges associated with upholding both the intrinsic stretchability and self-healability of RRAM remain to be resolved.

**Fig. 1:**
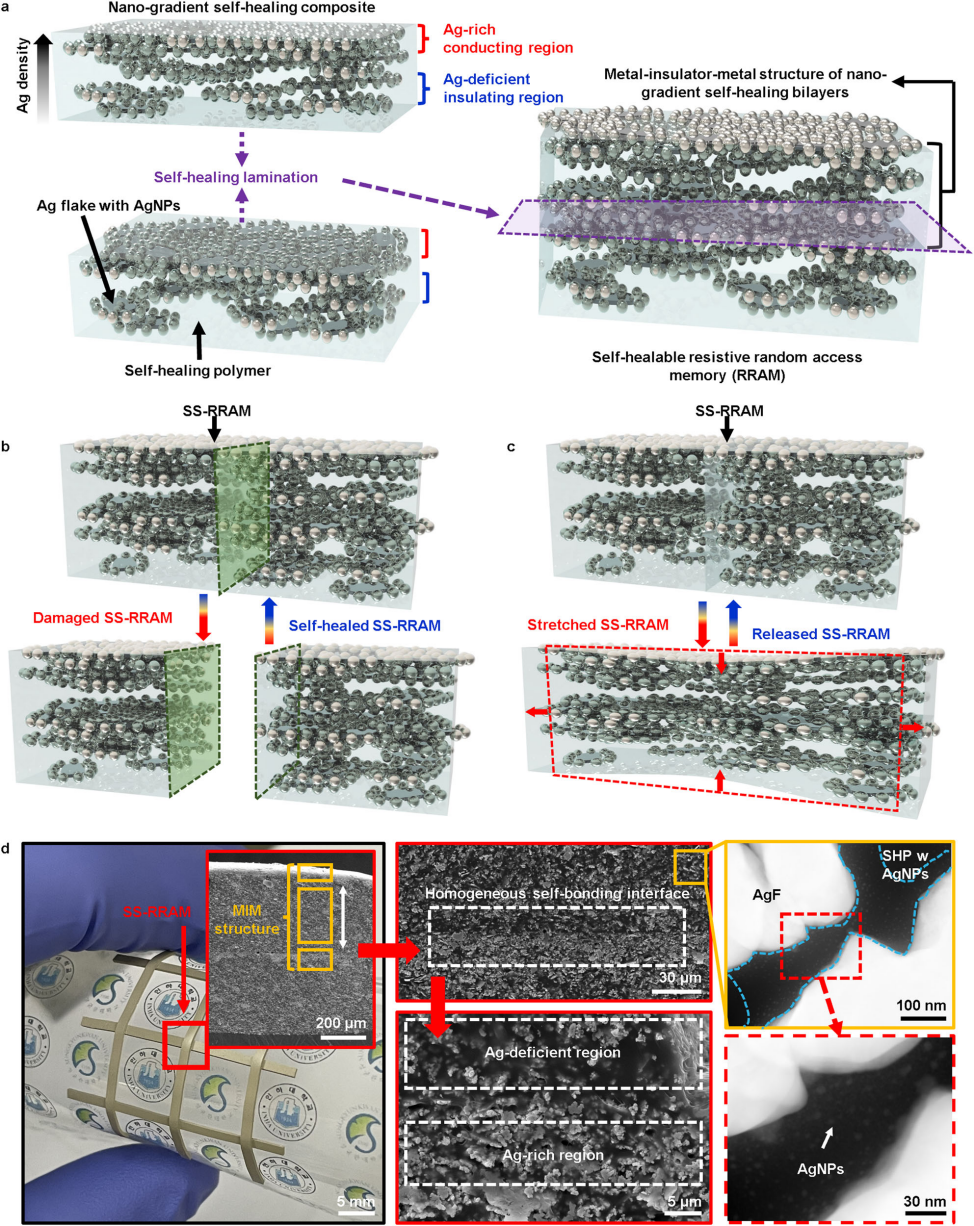
Schematic illustration of a Ag-GN film and fabrication of SS-RRAM and photographic/electron microscopy images of SS-RRAM. **a** Schematic diagrams of a Ag-GN film and its fabrication process by self-healing lamination. Ag-deficient region of the upper Ag-GN film laminated to the Ag-rich region of the bottom film, resulting in a metal–insulator–metal structure. **b** Schematic illustration of the self-healing process of the SS-RRAM. **c** Schematic illustration of a SS-RRAM presenting intrinsic stretchability. **d** Photographic image of a 3 × 3 SS-RRAM array (left) and SEM image showing a cross-sectional view of the MIM structure (inset left). SEM image of the self-bonding interface region (upper middle) and magnified SEM image of Ag-deficient/Ag-rich region (lower middle). TEM images of Ag flakes (upper right) with AgNPs (lower right).

Herein, we report the development of the self-healing stretchable resistive random-access memory (SS-RRAM) fabricated by allowing two Ag-gradient nanocomposites (Ag-GN) films to be a bilayer through the self-healing (Fig. 1 and Supplementary Fig. S1). These films are composed of a tough self-healing stretchable polymer (SHP; polydimethylsiloxane (PDMS)−4,4’-methylenebis(phenylurea) (MPU)_0.4_-isophorone bisurea units (IU)_0.6_) and silver micro-/nanoflakes (AgFs) (Fig. 1 and Supplementary Fig. S2). The unique structure of the Ag-GN was the spontaneous outcome of the drying process after drop casting the AgF composite solution with low viscosity onto the handling substrate. This asymmetric Ag distribution shows that the average resistance value (~10 Ω) at the bottom is much lower than that at the top. Ag flakes are prone to be concentrated and aggregated in the lower section of the Ag-GN film because of their higher density/interaction than SHP in the solvent and the gravitational effect of the drying process. Compared to the in-plane conducting path in the upper section, the resistance value (~1 TΩ) at the vertical path between the upper and lower sections is also high, indicating that resistive switching occurs in the insulating region of the SS-RRAM. The SS-RRAM shows typical unipolar resistive switching (URS) behavior where the high- and low-resistance states (HRS and LRS) are dominantly governed by Schottky emission/hopping and Ohmic conduction mechanisms, respectively. In addition, the SS-RRAM has a high on/off ratio of ~10^5^ (maximum and minimum ratio values of ~10^5^ and ~10^9^), stable electrical durability (cyclic electrical endurance of 500 cycles and data retention of ~50 h), and can be stretched up to 100% strain even after both cutting/reconnecting processes and heat-accelerated recovery (30 min at 60 °C). Based on its healing properties, the SS-RRAM can be used in reconfigurable modular electronics, potentially resulting in the realization of user-customizable electronic products. As a proof of concept, we demonstrated a reconfigurable device where a 1 × 4 array of SS-RRAM can be reconstructed to a 2 × 2 array without electrical malfunction. To highlight the self-healing ability of the SS-RRAM, we demonstrated stable data storage of cardiac signals^25^, damage-reliable memory triggering system using a triboelectric nanogenerator^26–29^ (TENG), and touch sensing via pressure-induced resistive switching.

## Results

### General description of the self-healing stretchable resistive random-access memory (SS-RRAM)

Intrinsically stretchable and self-healing insulating composite materials with resistive switching properties have not been reported yet because of the challenges associated with the unstable formation and/or reformation of electrically percolative pathways inside the dynamically cross-linked networks. Realization of the intrinsically stretchable and self-healing RRAM device has the following prerequisites: (i) self-reconstruction of the nanoscale conducting fillers in a dynamically cross-linked polymer with a low glass transition temperature (*T*_g_), (ii) stronger interaction among the individual conducting fillers than with polymers for robust formation of the percolation path (LRS), and (iii) an insulating layer in which the conducting fillers have the optimal density for precisely controlled resistive switching. To meet these requirements, we developed an asymmetric structure comprising a self-healing stretchable Ag-GN film with both conducting and insulating interfaces, as shown in Fig. 1a. The Ag-GN film, which consists of Ag flakes with in-situ formed Ag nanoparticles (AgNPs) and SHP with low *T*_g_, is similar to that in the previous report^30^ (Supplementary Fig. S3). However, previously, the conducting nanocomposite with uniformly distributed Ag flakes/AgNPs was only intended for the development of an “electrode” of which the top and bottom interfaces are both highly conductive. In this work, the in-situ formation of a resistive switching layer based on the Ag density gradient structure is a key switching element for realizing SS-RRAM. The two as-prepared Ag-GN films were bonded via the self-healing process at room temperature to form a metal–insulator–metal (MIM) structure. Details of the fabrication process are provided in Supplementary Note #1 and Supplementary Fig. S4. As expected, the SS-RRAM engages in spontaneous healing and can even be intrinsically stretched owing to the dynamic multivalent hydrogen bonding and low *T*_g_ of the SHP matrix (Fig. 1b and c). Cross-sectional scanning electron microscopy (SEM) and transmission electron microscopy (TEM) images of the SS-RRAM are shown in Fig. 1d. The 3 × 3 SS-RRAM arrays were simply fabricated by exploiting the self-healing property mentioned above. The magnified SEM images show the asymmetric structure of the laminated Ag-GN bilayer. Furthermore, the TEM images show that AgNPs were formed around the Ag flakes. The formation of AgNPs is known to contribute to the electron transport mechanism in the polymeric insulating layer^31,32^. These MIM structures are capable of acting as SS-RRAMs. Specifically, resistive switching can occur in the Ag-deficient insulating region sandwiched between the top and bottom Ag-rich conducting layers. Further, resistive switching under tensile stress can be stably performed by efficiently dissipating the strain energy of the Ag-GN cell itself and/or interconnects. Details of the electrical characteristics and modular/bio-signal data storage/self-healing demonstrations of the SS-RRAM are presented in the following figures.

### Observation and durability of unipolar resistive switching of the SS-RRAM

**Fig. 2:**
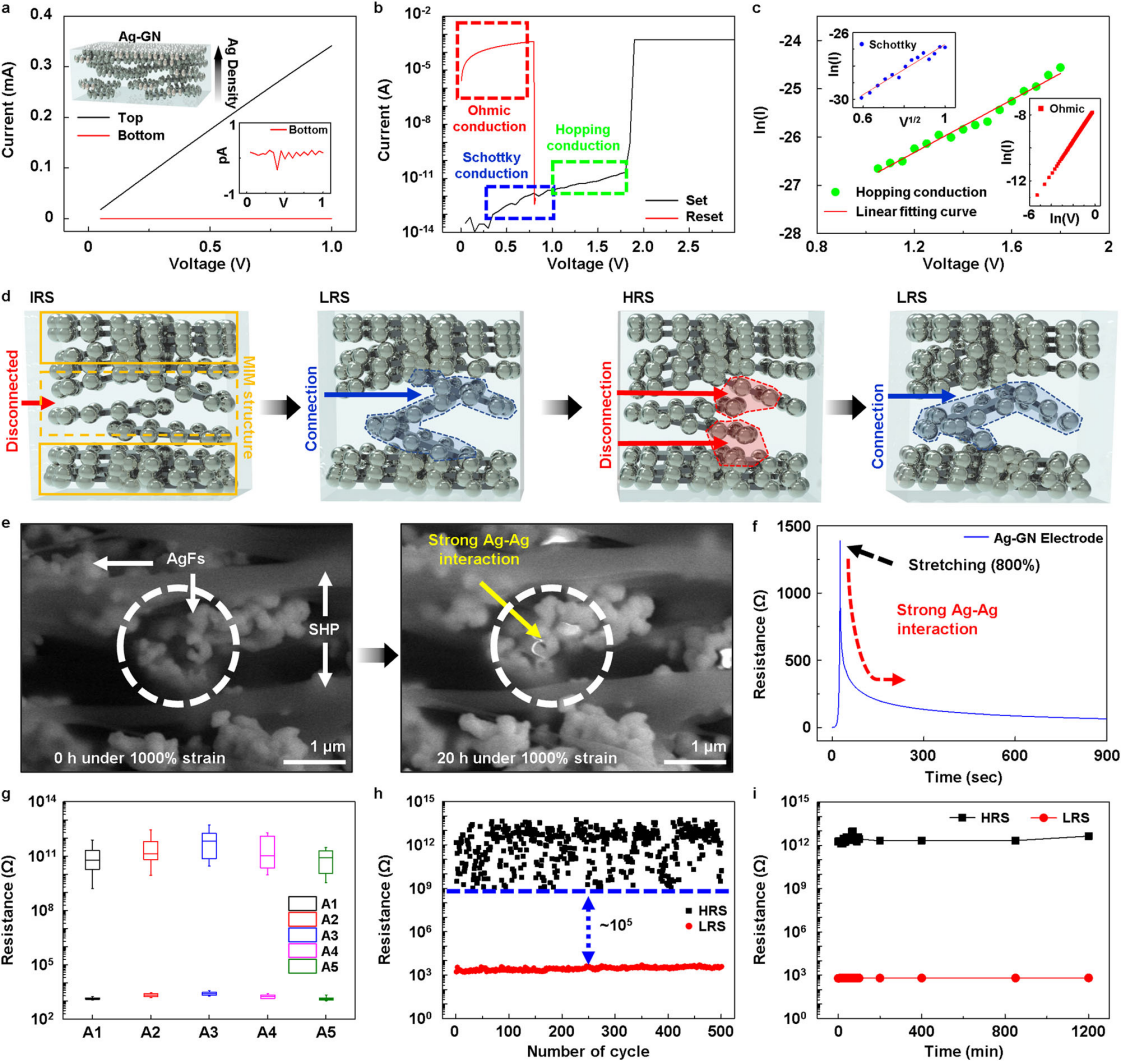
Electrical characterization and conduction mechanism of fabricated SS-RRAM. **a** *I*–*V* testing of a different region of the Ag-GN film. The upper inset shows the density gradient feature of the Ag-GN film with the Ag-rich region at the top and the Ag-deficient region at the bottom. The bottom inset shows the *I*–*V* curve at the picoampere scale. **b** Current–voltage characteristic of the SS-RRAM, which exhibited URS behavior. **c** Fitting curves of HRS and LRS regions for the analysis of the conduction mechanism. Green and blue dots represent the *I*–*V* characteristics of the HRS region (blue and green dashed region in Fig. 2b), which corresponded with Schottky emission and hopping conduction, respectively. The inset shows the *I*–*V* curve of the LRS region (red dashed region in Fig. 2b), which exhibited Ohmic behavior. **d** Illustration depicting the formation and rupture of the conduction path in each resistance state. **e** Reconstruction of AgFs in the SHP matrix under 1000% strain. **f** Electrical dynamic reconstruction of Ag-GN. **g** Distribution of HRS/LRS resistance in various cell sizes. The sizes of A1–A5 were 1 × 1, 1.5 × 1.5, 2 × 2, 2.5 × 2.5, and 3 × 3 mm^2^, respectively. **h** Electrical cyclic switching durability up to 500 cycles. **i** HRS and LRS retention performance of a SS-RRAM for 1200 min.

The electrical behavior in both the Ag-rich and Ag-deficient regions of the Ag-GN (Fig. 2a) was investigated by conducting current–voltage (*I*–*V*) measurements. The upper Ag-rich region is highly conductive (of the order of a few ohms), and the Ag-deficient region is almost insulating (~1 TΩ). This structure confirms the formation of asymmetric Ag-GN interfaces in the SS-RRAM when two of Ag-GNs form a bilayer. The resistive switching of the SS-RRAM was studied by measuring the *I*–*V* characteristics. A positive step bias was applied to the Ag-rich conductive upper layer of the SS-RRAM while connecting the lower Ag-rich layer to the ground (Fig. 1a). The SS-RRAM exhibited typical unipolar switching behavior, as shown in Fig. 2b. The change from the HRS to the LRS, termed “Set” and the reversal from the LRS to the HRS, termed “Reset” were observed at 1.9 and 0.815 V, respectively. To verify the mechanism underlying the conduction of the SS-RRAM, we analyzed its current–voltage characteristics. In the HRS of the SS-RRAM, *I*–*V* curves was mostly found to correspond with Schottky emission and hopping conduction. As shown in Fig. 2b and c, the *I*–*V* data are a good fit with the *V*^1/2^–ln(*I*) and *V*–ln(*I*) plots, which correspond to the Schottky and hopping conduction mechanisms, respectively^33^. The LRS of the SS-RRAM clearly exhibited Ohmic conduction behavior, as expected. Based on these electrical analyses, we propose a possible scenario for resistive switching of the SS-RRAM (Fig. 2d). The as-fabricated SS-RRAM cell is initially in the HRS, showing that the Ag flakes with the AgNPs in the Ag-deficient region are scattered and disconnected (Fig. 2d, 1st step). In the HRS, prior to the Set process, charge transport is presumably initiated by Schottky emission and accelerated by hopping/tunneling conduction. Once the DC bias is sufficient and reaches the Set voltage, the disconnected Ag flakes with AgNPs at the resistive switching region in the Ag-GN bilayer can be physically connected by forming conductive filament paths (Fig. 2d, 2nd step). Specifically, the field-driven formation of conducting paths in the SS-RRAM is in line with a previous report^31^. These paths are found to be reliably maintained because the Ag–Ag interaction is far stronger than that of Ag-SHP, even in the dynamic polymer matrix^30^. The resistance of the conductive filaments ranges from 0.1 to 4 kΩ. During the transition from the HRS to the LRS under Reset bias, the conductive filaments might be ruptured by the Joule heating process (Fig. 2d, 3rd step). The thermal imaging analysis of the switching process is presented in Supplementary Fig. S5. Application of the Set bias to the SS-RRAM cell enables the broken paths to be reconnected (Fig. 2d, 4th step). To support our assumption that the filamentary conduction path can be stable and maintained due to the strong Ag–Ag interaction, we demonstrated in-situ SEM analysis of the Ag-GN film (at 1000% strain) at 0 h (left SEM) and 20 h (right SEM) under 1000% strain (Fig. 2e). As direct evidence, this analysis shows that the separated AgFs are spontaneously aggregated, meaning that conducting pathways in the SS-RRAM can be stably maintained. Fourier-transform infrared spectroscopy (FTIR) data also showed that AgFs have no chemical reactions with the SHP (Supplementary Fig. S6). Further, when we suddenly stretched the Ag-GN film to 800% strain, the increased resistance values were observed to be significantly decreased without any external stimuli (Fig. 2f). This reconstruction of AgFs in the SHP matrix is also supporting what we suggested. Furthermore, the *I*–*V* characteristics of the SS-RRAM with different cell areas were also investigated. As shown in Fig. 2g, the resistive switching performance of the SS-RRAMs did not depend on the corresponding cells. This result indicates that resistive switching of the SS-RRAMs is highly related to the formation of conducting filaments^6^. In addition to the observation of resistive switching of the SS-RRAM, to confirm the electrical durability, the electrical cyclic endurance (500 cycles) of the SS-RRAM was investigated (Fig. 2h and Supplementary Fig. S7). The data retention performance (1200 min) of HRS and LRS at room temperature was also reliable (Fig. 2i). Further, the URS characteristics of the SS-RRAM devices under various temperature values of 30, 40, 50, and 60 °C were almost identical while showing the high electrical uniformity (Supplementary Figs. 7a and S7b). Specifically, the on/off ratio (*R*_HRS_/*R*_LRS_) larger than ~10^5^ was stably maintained up to 3000 mins even at 60 °C without any deviation of *I*–*V* curves and malfunctions (Supplementary Fig. S7c). Such thermal stability is fully compatible with long-term skin (37.5 °C) tissue interfacing.

To discuss the importance of the spontaneous formation of the Ag-GN structure for the stable resistive switching operation in the SS-RRAM, we compared the Ag density gradient structure-based SSRAM to two different devices using two types of nanocomposites: (i) spin-coated SHP thin film (thickness of SHP film was controlled by stacking each of the layers ranging from 1 to 3 using the self-healing property) sandwiched between the top and bottom Ag-GN electrodes, (ii) excessively phase-separated AgF-rich/-deficient composite embedded in top and bottom Ag-rich electrodes (Supplementary Figs. S8 and S9). First, we prepared the insulating SHP thin film embedded in the electrodes (Supplementary Fig. S8). The fabrication process began with spin-coating the SHP solution (1 g/CHCl_3_ 10 ml) onto the OTS-functionalized SiO_2_ wafer to form the ultrathin-insulating SHP film. After finalizing the drying process, the insulating SHP film was bonded to the surface of the Ag-rich composite electrode via its self-healing property. After detaching it from the handling wafer substrate, insulating/Ag-rich layers were mounted onto the Ag-deficient composite electrode using an identical manner. The thickness of the insulating SHP film can be controlled by using the aforementioned transfer-printing method. From the SEM images, we can see that the insulating film thickness ranges from 3.2, 6.3, to 9.2 μm. To confirm the feasibility of the three structures for the RRAM, we analyzed their current–voltage characteristics. Even after applying 100 V (for initiating the forming process) to the individual cells, no electrical changes were observed. Second, as compared to Ag-GN structure, we fabricated the AgF-rich/-deficient composite with excessive phase separation by reducing its viscosity (7.2 g/CHCl_3_ 20 ml) (Supplementary Fig. S9c). As-fabricated layers were put together through the self-healing property. In the current-voltage curve, we cannot find any resistive switching characteristics compared to that of Ag-GN based SS-RRAM. Contrary to the low viscosity of the AgF composite solution, we increased its viscosity (7.2 g/CHCl_3_ 12 ml) and then fabricated the layered structure to confirm the electrical functionality (Supplementary Fig. S9a). Due to its high viscosity, AgFs were uniformly distributed ranging from bottom to top electrodes, leading to the electrical short circuit. These control experiments fully support the importance of the formation of Ag concentration gradient structure in the SS-RRAM.

In addition to the comparative study of the Ag-GN structure to others, we confirmed that areal and batch-to-batch uniformity of the SS-RRAM devices fabricated via the drop-casting process (Supplementary Fig. S10). First, we evaluated the resistance values of HRS and LRS in the 25 SS-RRAM cells (Supplementary Figs. S10a and S11a). The cumulative probability shows that each resistance state of the SS-RRAM devices was uniform. Further, its variation was negligible. Second, we prepared three different 4-inch wafer-scale composite batches and analyzed their electrical characteristics (Supplementary Fig. S10b). Each of the resistive switching performances was almost identical, showing that our fabrication process is reliable.

Besides, we confirmed the electrical disturbance in the 5 × 5 SS-RRAM array (Supplementary Fig. S11). We sequentially programmed the individual cells as HRS in the 5 × 5 SS-RRAM array (Supplementary Fig. S11a, blue dashed boxes corresponding to the HRS RRAM cells; red dashed box indicating the selected RRAM cell). After performing the Reset process in every RRAM cell measurement to prevent the undesired sneak currents, the selected RRAM cell was not electrically disturbed by the sneak currents (Supplementary Fig. S11b and S11c). Using this technique, we could fabricate a 25 × 25 SS-RRAM array (625 cells in the area of 729 mm^2^). using the Ag-GN interconnect with a minimum width of 500 μm (Supplementary Fig. S12). Although we fabricated such a complex array, a rectifying element such as a diode (or selector device) or transistor should still be required to perfectly prevent sneak paths in a high-density array. The current pattern resolution is relatively low, as compared to those of high-performance inorganic RRAM devices fabricated through the semiconducting fabrication processes^34^. Currently, an application of the photolithography/etching processes to the intrinsically stretchable and self-healing composite electrode is highly challenging. Because a high concentration of AgFs in the polymer matrix interferes with UV transmission to the polymer. In spite of the limitation issue, we tried to fabricate the Ag-GN film as small as possible using a stencil mask. Unfortunately, although we tried to fabricate thinner electrodes (~300 µm) than before (500 μm), we could not achieve the specific resistive switching structure, “Ag concentration gradient”, in the composite film due to the fast solvent evaporation of the AgF-SHP solution while moving the blade in the screen-printing process (Supplementary Fig. S13). In the future, we believe that photolithography-compatible composite materials would overcome the resolution limitation issue. If such issues were resolved even at a nano-scale level, the current resistive switching of the SS-RRAM should also be performed. To confirm the nanoscale resistive switching, we used an atomic force microscopy (AFM) tip coated with Ag film (Supplementary Fig. S14). The Ag-coated AFM probe as the Ag-rich electrode was contacted onto the Ag-deficient surface of the Ag-GN film. Its URS behavior was almost comparable to those of normal SS-RRAM devices, meaning that the Ag-gradient structure can be utilized as the active resistive switching layer in the nanoscale RRAM device (Supplementary Fig. S14b).

### Stretchability of the SS-RRAM

**Fig. 3:**
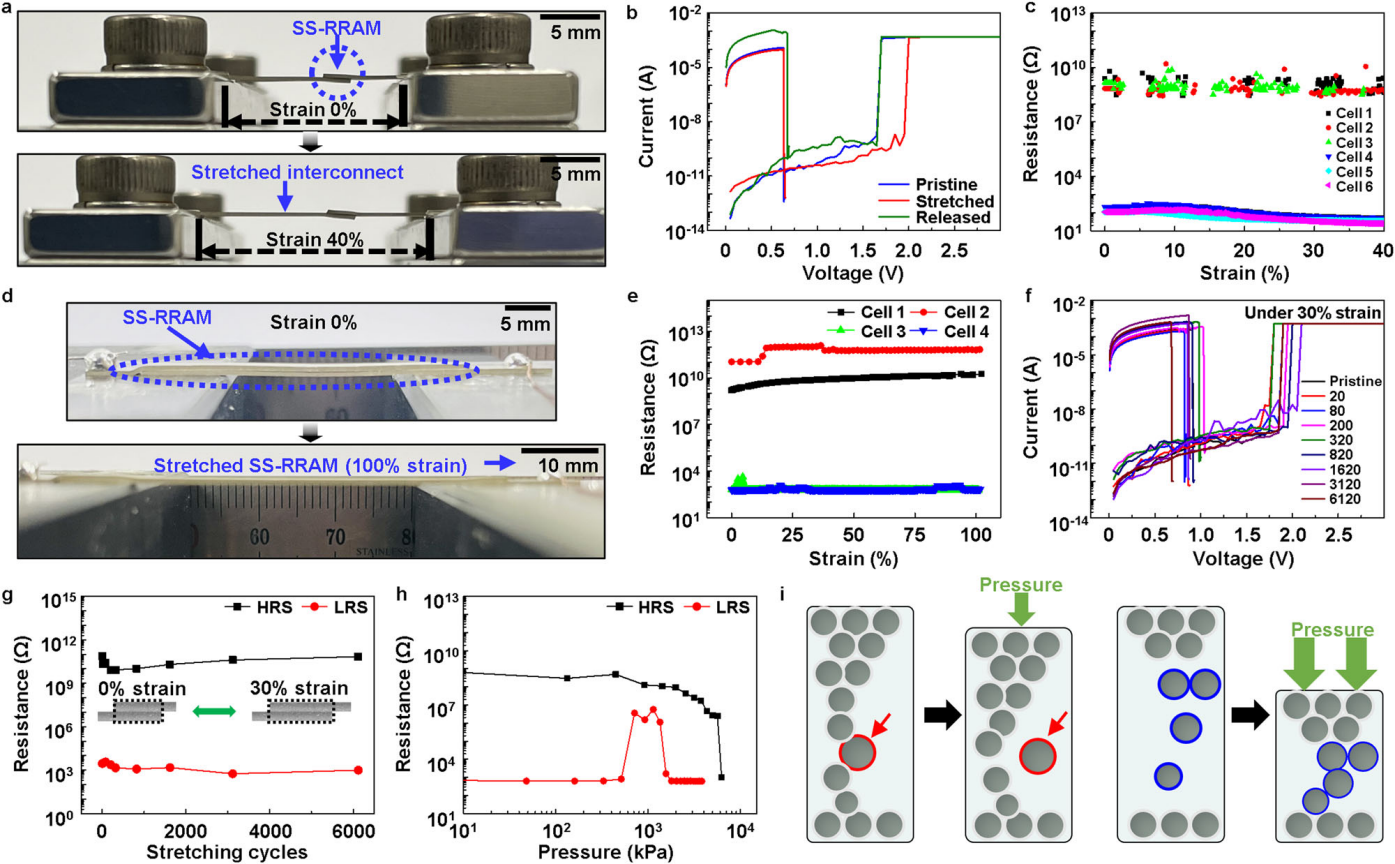
Stretchability of SS-RRAM. **a** Photographic image of the stretched SS-RRAM with strains of 0% (top) and 40% (bottom). **b** *I*–*V* characteristics of the pristine (blue), stretched (red), and released (olive) SS-RRAM. **c** Strain-resistance plot of the SS-RRAM during continuous elongation. Both the HRS and LRS exhibited minor changes in the resistance up to ~40% strain. **d** Images of the pristine and stretched SS-RRAM device in which a strain is applied solely on the cell region. **e** Plot of resistance of four SS-RRAM cells as a function of strain on cell region. **f** Electrical endurance of SS-RRAM device under 30% strain on cell region. **g** Switching stability of the SS-RRAM during the cyclic stretching test with the intentional application of 30% strain to the cell region. **h** Plot of resistance of SS-RRAM as a function of pressure. **i** Schematic diagrams depicting the change of each resistance state under compressive pressure.

The SS-RRAM should exhibit stable resistive switching even under tensile stress or when exposed to unexpected mechanical shocks. We confirmed the intrinsic stretchability of the SS-RRAM cell by stretching it until the strain reached 40% while measuring its *I*–*V* characteristics (Fig. 3a and b). The individual URS curves were uniformly observed for the pristine, stretched, and released states (Fig. 3b and Supplementary Fig. S15). Subsequent investigations included the stable retention performance of HRS and LRS in the six different SS-RRAM cells (1–3 cells corresponding to HRS; 4–6 cells indicating LRS) (Fig. 3c). Although the SS-RRAM showed reliable stretchability, most of the strain is occurring at the interconnect area (Fig. 3a). Again, the strain-dissipative property of the Ag-GN interconnect and the robust homogeneous self-bonded interface of the SS-RRAM allow it to be far more stable.

To clarify the intrinsic stretchability of the SS-RRAM without the strain-dissipative effects of the interconnect, we analyzed the resistive switching characteristics of the four different SS-RRAM devices with fully overlapped resistive switching areas (Fig. 3d and e). Figure 3e showed that LRS and HRS of the SS-RRAM devices were maintained while increasing strain values gradually up to 100%. The resistive switching of the SS-RRAM was stable even during the stretching cyclic test (more than 6000 cycles at a tensile strain of 30% solely on the cell region) (Fig. 3g and Supplementary Fig. S16). The stretchability of our soft SS-RRAM is enough to be applied to skin-like data storage or neuromorphic systems^35^.

Since the volatility of mechanical deformation of conventional RRAM has been a problematic issue for the realization of stretchable and wearable RRAM, it should be of significance to analyze the electrical behavior of our SS-RRAMs under mechanical compression. Figure 3h shows the retention of LRS and HRS SS-RRAMs under compressive mechanical pressure on the cell region. Both LRS and HRS SS-RRAMs started to be affected by relatively high enough values of compression, and the resistive switching occurred. In the case of LRS, it shifted to HRS firstly at ~512 kPa; it lasted at HRS to 1344 kPa and came back to LRS from 1560 kPa due to highly compressed spatial structure. Our SS-RRAM was based on a conduction path of filaments; the aligned AgFs of LRS squeezed, and some AgFs could be pulled out by mechanical compression; finally scattered AgFs were reconnected in a high compression regime (Fig. 3i). On the other hand, the resistance of HRS gradually decreased and finally shifted to LRS at 6200 kPa. The gradual decrease of resistance from the pressure of 1000 kPa can be explained by the deviation of distance among AgFs and AgNPs, which resulted in an increase in hopping electric current. The final switching of HRS to LRS occurred by percolative physical contacts of AgFs in a limited spatial structure driven by extreme compression. It should be noted that the first electrical failure induced by mechanical pressure was a higher value (512 kPa, change of LRS to HRS) than that of mild human activity like finger tapping (<50 kPa)^36^.

Such undesired switching phenomenon was also observed in an extreme stretching test of 300%. As the applied strain increased more than ~230%, the SS-RRAMs of LRS started to fail and switched to HRS (Supplementary Fig. S17a). It can be understood via induced pressure driven by a tensile strain. A finite-element method (FEM) simulation of the SS-RRAM stretching showed that compressive pressure of ~184 kPa was induced inside the polymer matrix at an in-plane tensile strain of 200% (Supplementary Fig. S17b, S17c and Note #2). The results were well matched with the previous compression experiment (Fig. 3h and i). In the case of HRS, it did not show any failure up to the strain of 300%. Considering compression results, it would last the internal pressure of ~6200 kPa, which was a much higher value than the induced one from the stretching. The trend of LRS and HRS also coincided with a previous study^37^. Nevertheless, if a proper pressure could be applied to the cell region of the SS-RRAM, one can utilize it for the switching of memory devices. We actually employed it as a means of switching presented later on.

### Self-healability and reconfigurability of the SS-RRAM

**Fig. 4:**
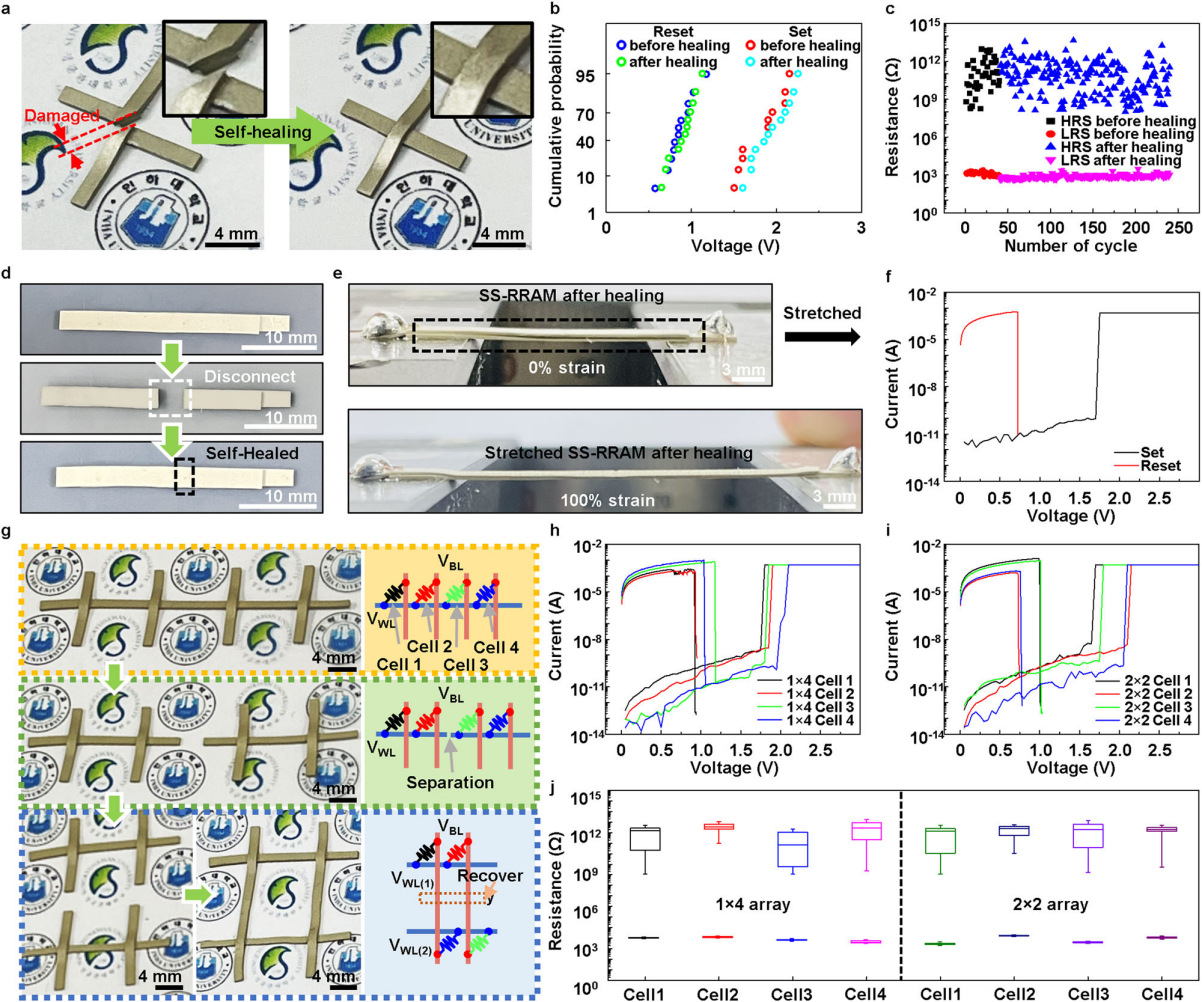
Self-healability and reconfigurability of SS-RRAM. **a** Photographic images of the SS-RRAM before (left) and after (right) the self-healing test accelerated by thermal heating at 60 °C for ~30 min. **b** Cumulative probability of Set and Reset voltage before and after the healing process. **c** Cyclic switching durability before and after the healing process. The resistance memory window was consistently wider than 10^5^ before and after healing. **d** Photographic images of a pristine (top), disconnected (middle), and self-healed (bottom) SS-RRAM, respectively. **e** Images of the stretched and self-healed SS-RRAM with a strain of 0% (top) and 100% (bottom). **f** *I*–*V* characteristics of the self-healed SS-RRAM under 100% strain. **g** Photographic images and the corresponding circuit diagrams of SS-RRAM array in reconfiguration process. The SS-RRAM array was rebuilt from a 1 × 4 to a 2 × 2 array. **h**, **i** *I*–*V* characteristics of the 1 × 4 and 2 × 2 SS-RRAM arrays. The electrical characteristics of each cell remained in the normal operating voltage window. **j** Resistance distribution of each cell in the HRS and LRS before/after the reconfiguration process.

Aside from the intrinsic stretchability of the SS-RRAM, its self-healability is also critical for skin-like memory or artificial synaptic devices. Figure 4a shows the self-healing ability of a damaged SS-RRAM under thermally accelerated conditions (60 °C) for 30 min. Herein, the self-healing performance with thermal actuation can be effectively accelerated without performance degradation^17,18^. After the self-healing process, the *I*–*V* curves of the pristine and healed SS-RRAM devices were almost identical (Supplementary Fig. S18). Further, the statistical distributions of the Set and Reset bias for 12 SS-RRAM cells before and after the self-healing process were confirmed to be uniform (Fig. 4b). An electrical cyclic endurance test to examine the durability of SS-RRAM (Fig. 4c) did not reveal any significant changes in the LRS and HRS of the SS-RRAM before and after the healing process while the on/off ratio remained stable. In addition, after healing, the SS-RRAM was stably stretched up to 100% strain while showing identical resistive switching performance (Fig. 4d–f).

Owing to the self-healing feature, the configuration of the memory array could possibly be rebuilt on instant demand. The images and corresponding schematic diagrams represent three types (a 1 × 4 array in the 1st row; two 1 × 2 arrays in the 2nd row; and a 2 × 2 array in the 3rd row) of SS-RRAM crossbar arrays (Fig. 4g and Supplementary Fig. S19). As shown clearly, this facile reconfiguration capability originates from the self-healing property of the SS-RRAM. The electrical properties of the SS-RRAM array were confirmed by conducting a statistical analysis of the *I*–*V* curves of the 1 × 4 and 2 × 2 SS-RRAM arrays (Fig. 4h–j). Details of the method we used to probe the reconstructed SS-RRAM array are presented in Supplementary Fig. S19 and Note #3. All the resistive switching data of the arrays before and after reconfiguration were comparable to those of the individual SS-RRAM cell. These results imply that the self-healing capability allows us to not only use a simple approach to construct the crossbar array structure, but also to re-construct it on instant demand.

### Stable data storage of cardiac signals using the reconfigurable 7 × 7 SS-RRAM array

**Fig. 5:**
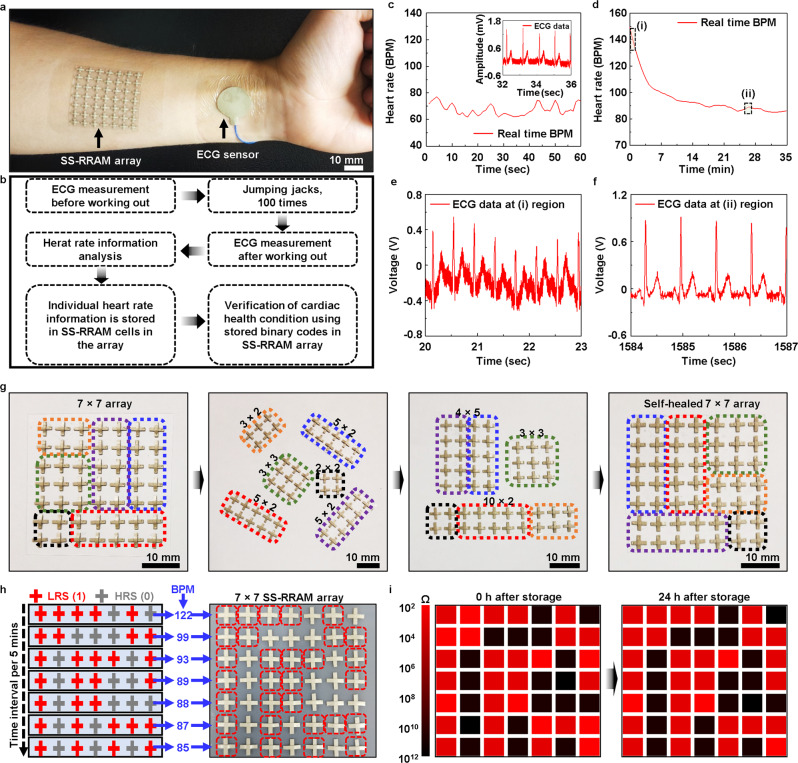
Stable data storage of ECG signals. **a** Image for 7 × 7 SS-RRAM arrays and ECG electrode mounted onto the wrist. **b** Flowchart of the sequential process for ECG measurement and data storage. **c** ECG signal measurement before and **d** after doing 100 jumping jacks. **e** Rapid recovery and **f** saturation regions in (**d**). **g** Images for reconfiguration and scalability of the SS-RRAM devices. **h** Schematic and the corresponding image of 7 × 7 SS-RRAM arrays after selective storage of individual BPM data. **i** BPM data storage map in 7 × 7 SS-RRAM array before and after 24 h.

As such, heart rates during working out are gradually increased. After finishing, the beats per min (BPM) recovered slowly. The trend of the BPM change can be a crucial indicator for precisely making a diagnosis of abnormal cardiovascular parasympathetic functions that normally occur after finishing the exercise^38^. To confirm the feasibility of the SS-RRAM for healthcare applications, we demonstrated the stable data storage of electrocardiogram (ECG) signals generated from the human body using an ECG electrode that has a low impedance value (65 Ω) at 79 Hz (Fig. 5 and Supplementary Fig. S20). Before starting with measuring electrophysiological signals, the 7 × 7 SS-RRAM array and ECG electrode were mounted onto the wrist (Fig. 5a). To improve skin conformability, we encapsulated the SS-RRAM array and ECG electrode using a transparent rubbery film (3M Tegaderm, USA) (Fig. 5a). The details of the experimental setup and sequential process for measurement of ECG signals are addressed in Fig. 5b and the section “Methods”. First, we measured the ECG signals (the BPM values ranging from 62 to 79) before working out (Fig. 5c). After doing 100 jumping jacks, the ECG signals were also recorded (Fig. 5d), showing the typical trend of the BPM decrease in a healthy body. Further, (i) for rapid recovery and (ii) for recovery saturation are comparable to those of the previous report^38^ (Fig. 5e and f). Such information should be stably stored in the SS-RRAM cells. Our memory device array can be reconfigured and rescaled due to its self-healing property (Fig. 5g). This unique characteristic potentially enables optimization of data storage capacity depending on the amount of bio-signal information. To store such information, each of the averaged BPM data (122, 99, 93, 89, 88, 87, and 85 every 5 min) within an interval of 5 min in total 35 min were individually transformed into the binary numbers (1111010, 1100011, 1011101, 1011001, 1011000, 1010111, and 1010101), respectively (Fig. 5h, left: schematic for RRAM cells with LRS (in red color) and RRAM cells with HRS (in gray color); right: the corresponding image with red dashed box that is Set as LRS). This data storage can be shown as an electrical resistance map for better readability (Fig. 5i and Supplementary Fig. S21). Such personal information was stably maintained after 24 h (Fig. 5i, right). This functionality can be further reliable against perspiration on the skin through the encapsulation of the hydrophobic SHP film onto the SS-RRAM device (Supplementary Fig. S22).

### Demonstration of damage-reliable memory triggering system using a triboelectric system

**Fig. 6:**
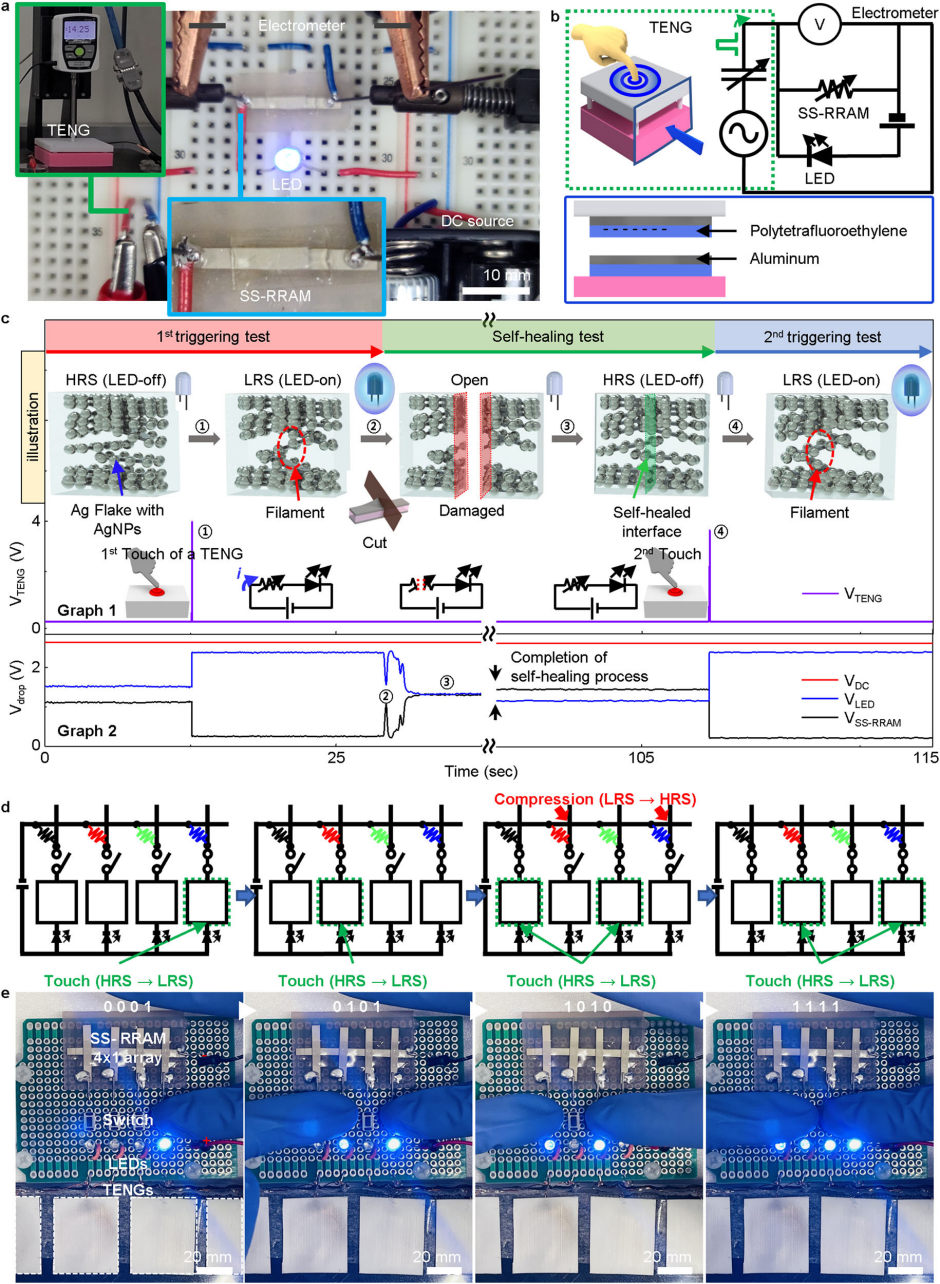
Demonstration of a damage-reliable memory triggering system using a triboelectric system. **a** Photographic images of the circuit components of an instant triggering system comprising SS-RRAM, TENG, LED, and DC battery. **b** Circuit diagram of an instant powered SS-RRAM triggering system and a scheme depicting information about the materials of the TENG. **c** In-situ time–voltage characteristic and schematic illustration of the triggering system. The voltage signals from the triboelectric device are shown in Graph 1; the voltage drops in the DC battery, LED, and SS-RRAM are presented in Graph 2. **d** Circuit diagrams corresponding to 4-bit number data storage. **e** Photograph image of 4-bit number of transition (storage/erase) from ‘0001’ to ‘1111’.

The soft and stretchable nature and even the self-healing properties of the SS-RRAM play a critical role in developing damage-reliable wearable memory devices. However, the power consumption of wearable devices is another crucial problem that would need to be overcome to allow long-term stable healthcare monitoring or to sustain the Internet of Things^39–41^. To address this challenge, we demonstrated a non-volatile memory triggering system capable of switching by instant energy harvested from TENG. The system consists of SS-RRAM, TENG, light-emitting diode (LED), and DC battery modules (Fig. 6a) in which the TENG consists of two conductors and one dielectric (Al-PTFE-Al) (Fig. 6b). The LED was employed as a visual indicator for the resistance states of the SS-RRAM. Note that the battery was solely utilized for powering the indicating LED rather than for switching the SS-RRAM. Prior to the integration of all the components, the electrical performance of the powering unit, that is, the TENG was examined first. In brief, the power output was set to the appropriate level by adjusting the operating height of the TENG^42^. Particulars of the working principle and power output performance of the TENG utilized in this demonstration are presented in Supplementary Figs. S23 and S24 and Note #4^43^. With optimal power management, the TENG triggered the resistive switching of our SS-RRAM where the HRS switched to the LRS. Optimization of the power selection for the TENG is shown in Supplementary Fig. S25 and described in Note #5.

All of the aforementioned components were integrated for the full visualization of the instant switching and self-healing features of our non-volatile memory system, as shown in Fig. 6b. Figure 6c presents illustrations of the in-situ voltage signals of each component. The SS-RRAM was initially at the HRS and remained in the integration. The LEDs also remained in the off state because the voltage signals from the DC battery were divided between the SS-RRAM in the HRS and LED according to the resistance ratio of these devices. Operation of the TENG by providing external mechanical input (i.e., finger tapping), delivered instant peak power to the SS-RRAM, thus forming an internal conductive filament path. The SS-RRAM then enters the LRS and can be regarded as a conductor in the circuit, enabling the blue LED to receive sufficient power from the battery and thus light up. To demonstrate real-time self-healing during operation, a razor blade was used to completely dissect the RRAM cell. This discontinued the power supply to the LED indicator, which was turned off again, confirming the open status of the circuit. Note that LRS shifted to HRS before and after the healing process, which resulted from a usual misalignment of conducting filaments during a manual cutting and healing process (Supplementary Fig. S26). Interestingly, we observed the extent of self-healing stepwise by monitoring the voltage drops across the SS-RRAM and LED. During the healing process, the voltage signal reflected the open status of the circuit and gradually stabilized to a certain value, which indicated the completion of the self-healing process. A 2nd triggering experiment was conducted to confirm the completion of the self-healing and recovery of the resistance state of the SS-RRAM. The SS-RRAM in the HRS again switched to the LRS as a result of the 2nd TENG triggering.

Inversely, switching of the SS-RRAM from LRS to HRS with a TENG could be somewhat challenging since it could deliver its power to devices with comparable impedance (~GΩ). SS-RRAMs at LRS have a relatively lower impedance (<4 kΩ) compared to TENG (>1 GΩ), and thus the Reset process using TENGs shall need an electronic circuit (or parts) to achieve so. The SS-RRAM was switched from LRS to HRS with a consisted RC circuit driven by a TENG (Supplementary Fig. S27). To circumvent a complex circuit formation, the two-way switching of SS-RRAM can be achieved by utilizing a combination of electrical and mechanical stimuli. Figures 6d and e exhibited a unique demonstration of two-way switching of SS-RRAMs in which a 4-bit memory device was presented. A finger tapping motion on a designated TENG was utilized as a power source in the Set process (Supplementary Fig. S28). And high compressive pressure (>500 kPa) on RRAM with a pointy pencil was utilized as the Reset process. Combining these, any given 4-bit binary number could be stored in a 1 × 4 SS-RRAM array and erased on instant demand. The demonstration showed a sequential change of 4-bit binary numbers (0001, 0101, 1010, and 1111). Notably, all switching of SS-RRAMs was solely powered by human activity such as touching a TENG or pressing with a pointy plastic pencil. It is a unique demonstration of real-time and on-demand data storage and ease of human activity, and it shows the potential of stretchable and wearable RRAM in future applications.

## Discussion

We report the first self-healable and stretchable RRAM, fabricated through the formation of the Ag-GN bilayer that has both conducting and insulating interfaces. The SS-RRAM comprised a self-healable/stretchable polymer matrix and conducting Ag-GN films. The region across which the gradient existed was employed as a switching layer for the SS-RRAM. The SS-RRAM based on the Ag-GN exhibited typical URS with a resistive window larger than 10^5^. The electrical properties of the SS-RRAM, including the endurance, switching type, and on-off ratio, were largely unaffected by mechanical stretching, moderate thermal energy, or cell-cut/healing processes. The self-healing ability of the interconnecting region revealed that the RRAM array was instantly reconfigurable. As a proof of concept, a 1 × 4 array was successfully converted to a 2 × 2 array. All cells were operated similarly, and newly connected bit- and word-lines were confirmed to function well before and after the conversion. Furthermore, the reconfigured SS-RRAM array enabled the stable data storage of electrophysiological signals generated from the human body. This self-healing property of the SS-RRAM is foreseen to become a key feature of future reconfigurable modular electronics or user-customizable electronic products. A real-time demonstration of this unique property of the SS-RRAM was conducted to visualize the operation of the self-triggering resistive switching system. Our findings are expected to provide a stepping-stone for the realization of user-customizable electronics and smart in vivo systems.

## Methods

### Fabrication of SS-RRAM

Amine terminated PDMS (Gelest, Mn = 5000, 100 g) and chloroform (Samchun, 400 mL) were stirred at 0 °C under N_2_ atmosphere. After 1 h, Triethylamine (Sigma Aldrich, 10 mL) was added to a flask. After stirring for 1 h, 4,4’–Methylenebis(phenyl isocyanate) (TCI, 2.0 g) and Isophorone diisocyanate (TCI, 2.7 g) were dissolved in chloroform (10 mL) and injected into a flask. After 30 min, the reaction temperature is gradually increased to room temperature and reacted for 4 days. After the reaction, Methanol (Samchun, 15 mL) was added to flask and stirred for 30 min. And reaction flask was concentrated to half of its and 60 mL of Methanol was added. After 30 min, the upper solution and viscous liquid were removed. 100 mL of chloroform was added and stirred to dissolve the product material. The dissolution, precipitation, and decantation were repeated 3 times. The product solution was concentrated in a vacuum evaporator and dried at room temperature for 2 days. The as-prepared SHP (2 g) was dissolved in 10 mL chloroform while stirring. After 30 min, to lower the viscosity of this solution, we added 6 mL chloroform to the SHP solution, and stirring was continued for 30 min. Then, Ag flakes (5.8 g) were slowly added to 16 mL of the SHP solution while stirring for 30 min. Then, the Ag flake-SHP solution was drop-casted onto an octadecyltrimethoxysilane-functionalized silicon dioxide/silicon wafer substrate. After one day, the in-situ formation of Ag nanoparticles around the Ag flakes was finalized. The as-fabricated composite was the Ag-GN film. Because these Ag-GN films are freestanding, they can be detached from the handling wafer. The detached film was cut into two Ag-GN films using a razor blade. The conducting surface (bottom surface) of the first Ag-GN film was laminated onto the insulating surface of the second one using a self-bonding assembly at room temperature. This Ag-GN bilayer structure forms the SS-RRAM.

### Electrical characterizations of RRAM

*I*–*V* measurements were conducted using a semiconductor analyzer (SCS-4200, Keithley) with tungsten probe tips (model: T20, straight needle shape, tip-end diameter of 1 µm). The liquid metal (Gallium–Indium eutectic, Ga 75%, In 24.5% >99.99% trace metals basis, Sigma Aldrich) was utilized to lead out SS-RRAM from the tungsten tips to prevent undesired damage to the switching region of the SS-RRAM. The LABVIEW (National Instrument) program was customized to control the SCS-4200. Initially, the SS-RRAM was placed in a probe station (Tp-802x manual Probestation, Trip). A DC bias was applied to the top electrode of the RRAM while the bottom electrode was grounded. In the Set process, a compliance current of 500 µA was pinned to prevent undesired strong electrical breakdown or the formation of irreversible filaments. In the Reset process, the compliance current was limited to 0.1 A. The resistance in the HRS and LRS during the retention test was measured by applying a voltage of 0.4 and 0.15 V to the conducting upper layer of the polymer, respectively.

### Reconstruction of AgFs in the strained self-healing polymer for explaining robust AgF–AgF interaction

Electrical dynamic reconstruction of Ag-GN was performed using a stretching machine comprising a stretching stage with a step motor, controller (SMC-100, Ecopia), and a laptop. (Motorized X-translation stage, Jaeil Optical Corp.). To analyze the electrical dynamic reconstruction, we prepared the Ag-GN film (length of 3 mm, width of 3 mm, and thickness of 0.3 mm). The Ag-GN film was suddenly stretched up to 800% strain with a fast stretching rate of 1 mm per second to electrically degrade its conductivity (for the formation of the broken AgF aggregates). And then, the resistance values of the strained Ag-GN film were monitored, showing a gradual decrease in resistance of the Ag-GN film. Such electrical change originates from the spontaneous reconstruction of the AgFs in the strained SHP matrix.

### Current–voltage measurement in nanoscale

The conductive atomic force microscopy was conducted via customized AFM (Model: XE7, Park Systems) equipped with a sourcemeter (Keithley 2410, Keithley) and a laptop. The AFM cantilever (25pt-300B, Park Systems) was coated with Ag thin film using the sputter. The Ag-coated tip was contacted to the top of the Ag-GN film. The LABVIEW (National Instrument) program was customized to apply a voltage through the sourcemeter. In the current–voltage measurement, the positive bias was applied to the Ag-coated AFM tip, whereas the bottom of Ag-GN film was grounded. The compliance current was set as 500 µA and 0.1 A under the Set and Reset operations, respectively.

### Device preparation for the intrinsically stretchable SS-RRAM with short and long interconnects

The stretching test of the SS-RRAM was conducted by applying a stretching speed of 6 μm/s, while analyzing its electrical behavior using the semiconductor analyzer (SCS-4200). For analyzing the strain-dissipative effect of the SS-RRAM, we prepared the SS-RRAM with two long interconnects (total length of the interconnect: 15 mm, cell size: 1 × 1 mm^2^) (Fig. 3a–c). Further, the dependency of resistive switching performance of the SS-RRAM devices on the cell area was analyzed (Figs. 3d–g). To confirm the intrinsic stretchability of the SS-RRAM, we also prepared the SS-RRAM with short electrodes (total length: ~18 mm, cell size: ~1 × 15 mm^2^).

### Mechanical compression test

Compression tests were taken using compression and electrical measurement systems. The step motor controller (SMC-100, Ecopia) and force gauge (M2-10, Mark-10) were used to apply and measure the loads to a single SS-RRAM cell (cell size: 5 × 5 mm^2^). Simultaneously, the resistance values were measured under an application of bias of 0.4 V using a semiconductor analyzer (SCS-4200, Keithley) under different loads, which was controlled by the LABVIEW program.

### Electrocardiogram (ECG) measurement

A surface 3-lead electrocardiography was used to measure the real-time heart beats per minute (BPM). The Ag-GN film, acting as a surface electrode of the ECG sensor was attached to the wrist. The electrode was tightly mounted onto the skin using a transparent rubbery film (3M Tegaderm, USA). To measure the ECG signals precisely, three ECG electrodes were mounted on the right hand (reference), left hand (ground), and left leg (active), respectively. The BPM values were analyzed before and after doing 100 jumping jacks. Such measurement was performed for 35 min after doing 100 jumping jacks. The average BPM data were calculated every 5 min. ECG signals were obtained at a sampling rate of 1 kHz using a data acquisition device (Powerlab 8/35, AD Instruments, Australia) and an amplifier (Bio Amp, AD Instruments, Australia). The obtained signals were processed using LabChart 8 Pro (AD Instruments, Australia) software. The authors obtained Institutional Review Board (IRB) approval (No. SKKU 2022-05-015) from Sungkyunkwan University for real-time measurement of the ECG signals.

### Reconfiguration of 7 × 7 SS-RRAM array using self-healing property

The individual 49 SS-RRAM cells (area of a single memory is 4 × 4 mm^2^) in the 7 × 7 arrays were fabricated on the self-healing substrate. The 7 × 7 arrays were divided into six small ones (2 × 2, 3 × 2, 3 × 3, and three 5 × 2). The individual SS-RRAM arrays were reconfigured as 4 × 5, 3 × 3, and 10 × 2 ones through the self-healing process. Using the same strategy, the separate arrays were combined as the 7 × 7 SS-RRAM device. The electrical characteristics of the reconfigured 7 × 7 SS-RRAM array were analyzed by using the semiconductor analyzer (B1500A, Keysight).

### Demonstration of a damage-reliable memory triggering system using a triboelectric system

(1) A damage-reliable memory triggering system: The system comprised a triggering RRAM and an LED battery to serve as an indicator. The triggering subsystem comprised a step motor controller (SMC-100, Ecopia), force gauge (M2-10, Mark-10), and triboelectric nanogenerator (TENG) mounted on a 3D printed supporting stage. Polytetrafluoroethylene (PTFE) film and Al film were employed as negative and positive triboelectric materials, respectively. The active contact area of the TENG was ~25 × 25 mm^2^. The electrical power output from the TENG was measured using an electrometer (6514, Keithley) and an oscilloscope (MDO3024, Tektronix) with the customized LABVIEW program. The electrometer was used on a high impedance setting (>200 TΩ) to measure the voltage drop in the SS-RRAM.

(2) Two-way switching demonstration of 4-bit binary numbers: The system comprised a single electrode TENG, 1 × 4 SS-RRAM arrays, designated four LEDs, and a battery. The size of a single electrode TENG was 20 × 25 mm^2^. PTFE film and nitrile globe were employed as negative and positive triboelectric materials, respectively. The Set process was conducted by finger tapping the TENG with a nitrile globe. The Reset process was performed by pressing the cell region of the SS-RRAM with a pointy pencil (>500 kPa).

## Supplementary information

Supplementary Information

## Data Availability

All data that support the findings of this study have been included in the main text and Supplementary Information. The source data of the main figures are provided with this paper. Any additional materials and data are available from the corresponding authors on reasonable request Source data are provided with this paper.

## Source data

Source Data
