# Supplementary material for: Reversible electrical percolation in a stretchable and self-healable silver-gradient nanocomposite bilayer
Source: Nat Commun. 2022 Sep 5;13:5233. doi: 10.1038/s41467-022-32966-x (PMC9445036; doi:10.1038/s41467-022-32966-x)
Supplement: Supplementary file 1 — Supplementary Information [file 41467_2022_32966_MOESM1_ESM.pdf]

Reversible electrical percolation in a stretchable and self-healable silver-gradient nanocomposite bilayer

Jinhong Park[1,2†], Duhwan Seong[3,4†], Yong Jun Park[2†], Sang Hyeok Park[2], Hyunjin Jung[3,4], Yewon Kim[3,4], Hyoung Won Baac[3], Mikyung Shin[4,5], Seunghyun Lee[6], Minbaek Lee[1,2]*, and Donghee Son[3,4,7]*

[1]*The Institute for Basic Science, Inha University, Incheon 22212, Korea*
[2]*Department of Physics, Inha University, Incheon 22212, Republic of Korea*
[3]*Department of Electrical and Computer Engineering, Sungkyunkwan University, Suwon 16419, Republic of Korea*
[4]*Center for Neuroscience Imaging Research, Institute for Basic Science (IBS), Suwon 16419, Republic of Korea*
[5]*Department of Intelligent Precision Healthcare Convergence, Sungkyunkwan University, Suwon 16419, Republic of Korea*
[6]*Department of Electronic Engineering, Kyunghee University, Yongin 17104, Republic of Korea*
[7]*Department of Superintelligence Engineering, Sungkyunkwan University, Suwon 16419, Republic of Korea*

[†]J. Park, D. Seong, and Y. J. Park contributed equally to this work.

[*]Corresponding authors. E-mails: daniel3600@g.skku.edu, mlee@inha.ac.kr

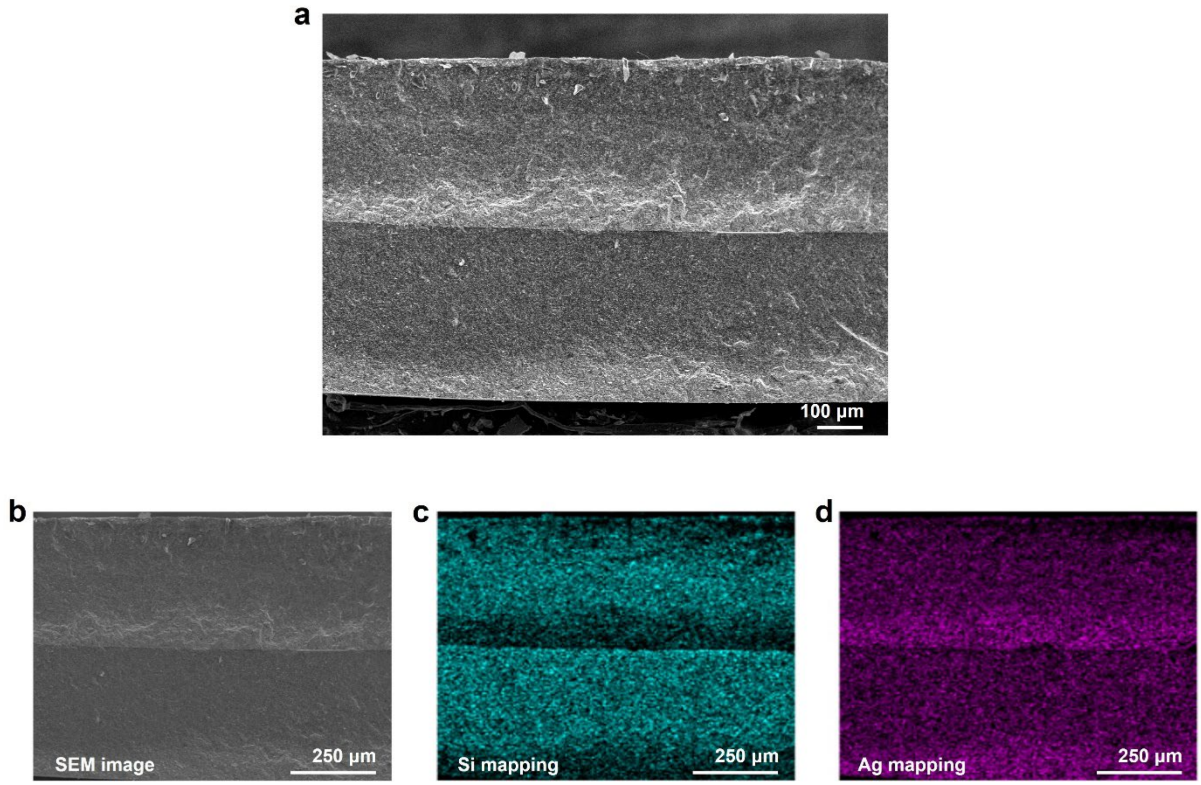

Supplementary Fig. S1 | Scanning electron microscopy (SEM) analysis of SS-RRAM. a, Cross-sectional SEM image of SS-RRAM. **b,** SEM image with low resolution. **c,** Energy dispersive spectroscopy (EDS) image of SS-RRAM for Si element**. d,** EDS image of SS-RRAM for Ag element.

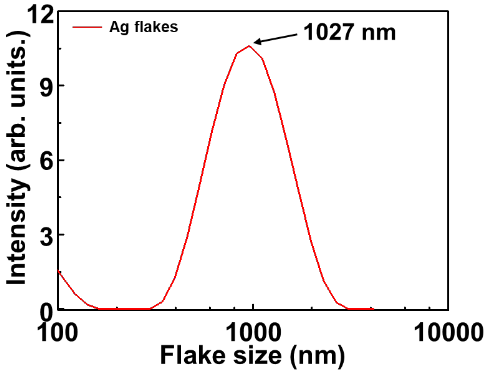

Supplementary Fig. S2 | Dynamic light scattering (DLS) analysis of Ag flakes. Plot of intensity of Ag flakes as a function of Ag flake size.

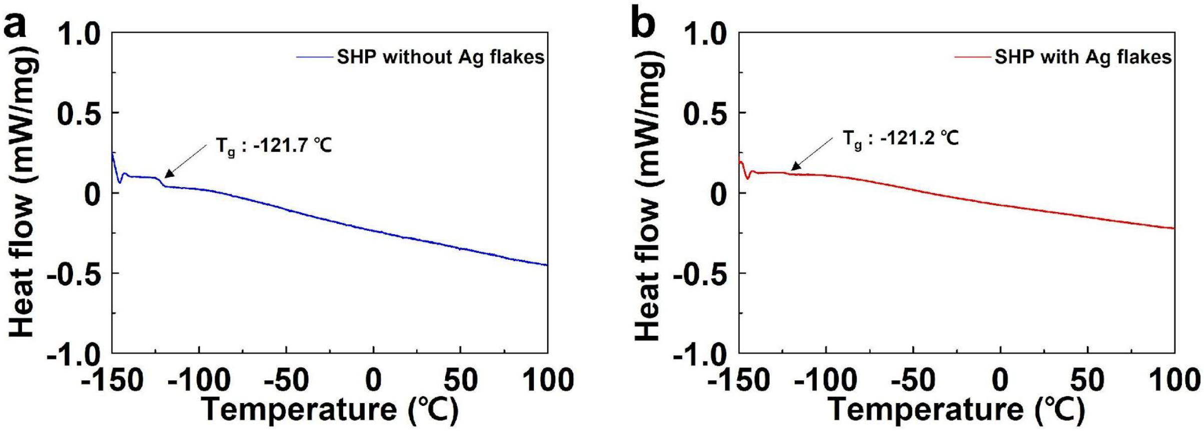

Supplementary Fig. S3 | Differential scanning calorimetric (DSC) thermograms. a, DSC data of pure self-healing polymer. **b,** DSC data of AgF nanocomposite conductor.

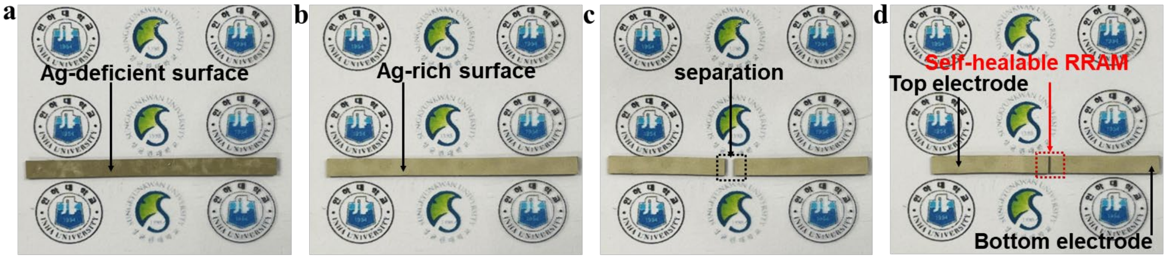

Supplementary Fig. S4 | Fabrication procedure of Self-healing and stretchable random-resistive access memory (SS-RRAM). a-c, The two AgF-SHP composite electrodes ((a), (b), and (c)) were prepared. **d,** The Ag-rich surface of AgF-SHP composite electrode was laminated onto the Ag-deficient one of the other composite electrode.

Supplementary Note #1

The fabrication procedure of SS-RRAM can be described as following steps: i) Prepare the long Ag-GN film. ii) Flip the Ag-GN film so that the Ag rich side electrode expose on the top. The top and bottom surface of Ag-GN film could be easily distinguished owing the difference in density of silver, as shown Fig. S1 i-ii. iii) Cut the long Ag-GN film to form two desired size of Ag-GN films. iv) Place the one Ag-GN film on top of the other. Thus the attached region between each Ag-GN film autonomously bonded through the self-healing process, in which dynamic multivalent hydrogen bonding and low glass transition temperature of SHP matrix were involved.

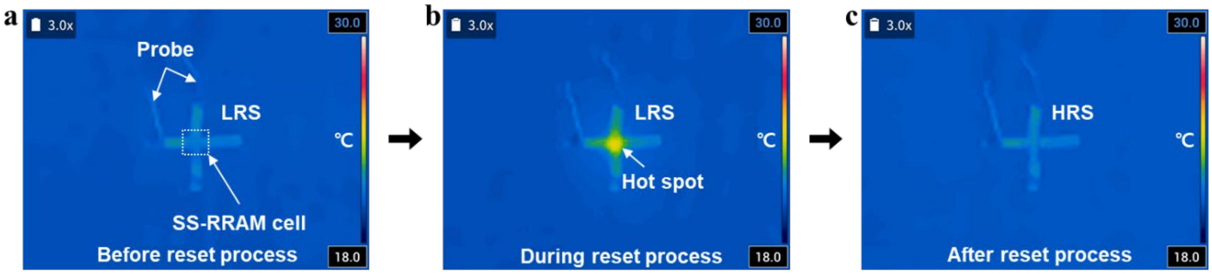

Supplementary Fig. S5 | Measurement of thermal image of SS-RRAM under joule heating process. a, Thermal mapping of ss-RRAM before applying reset process. **b,** Thermal mapping of ss-RRAM during reset process, showing the hot spot where joule heating occurs. **c,** Thermal mapping of ss-RRAM after joule heating process.

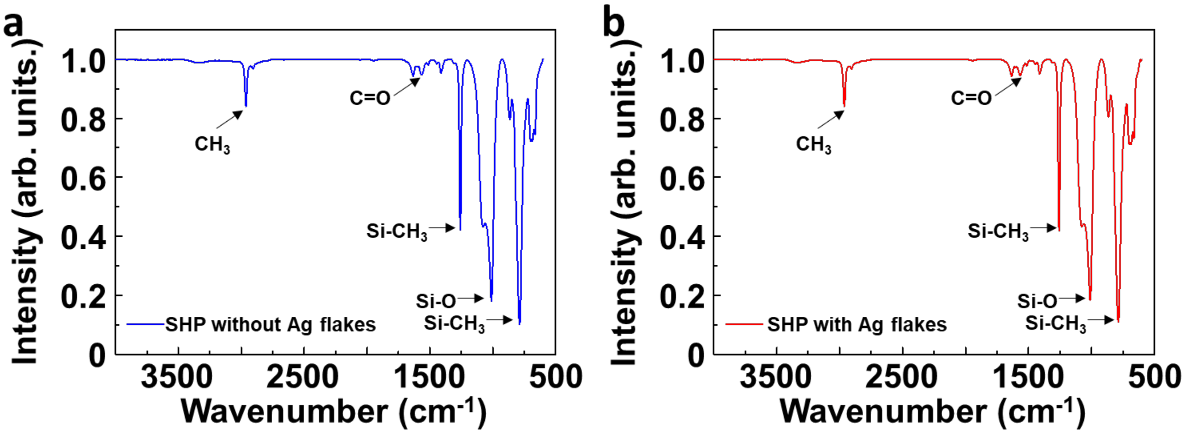

Supplementary Fig. S6 | Fourier-transform infrared spectroscopy (FTIR) data. a, FTIR data of pure self-healing polymer. **b,** FTIR data of AgF nanocomposite conductor.

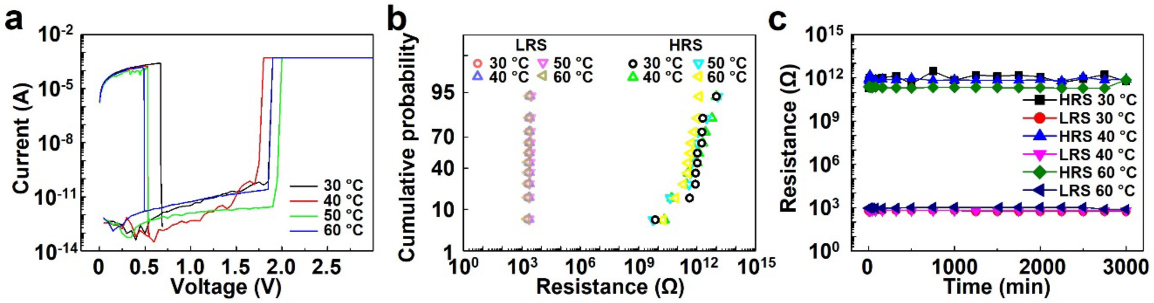

Supplementary Fig. S7 | Thermal stability and retention behavior of SS-RRAM with temperature of 30 °C, 40 °C, 50 °C and 60 °C. a, Current-Voltage characteristic of SS-RRAM with different thermal heating conditions. The black, red, green and blue lines represent I-V characteristics of SS-RRAM with temperature of 30 °C, 40 °C, 50 °C and 60 °C respectively. All I-V curves exhibited unipolar resistance switching behavior. **b,** Cumulative probability of SS-RRAM with different temperatures. **c,** Effect of temperature on the HRS and LRS data retention behavior.

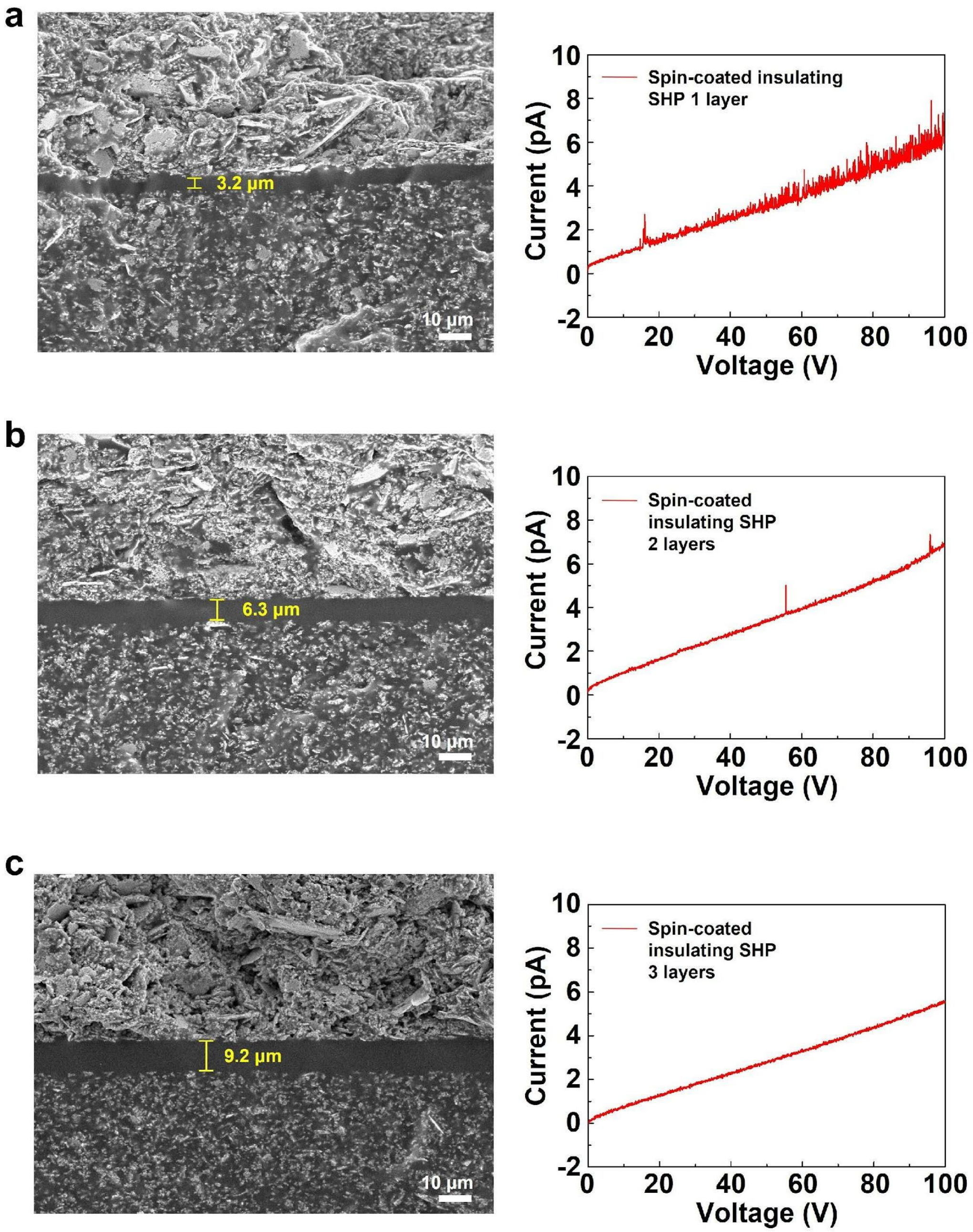

Supplementary Fig. S8 | SEM images and the corresponding I-V curves of the three capacitor structures comprising a spin-coated SHP layer. a, Capacitive structure using a single SHP layer. **b,** Capacitive structure using double SHP layers. **c,** Capacitive structure using triple SHP layers.

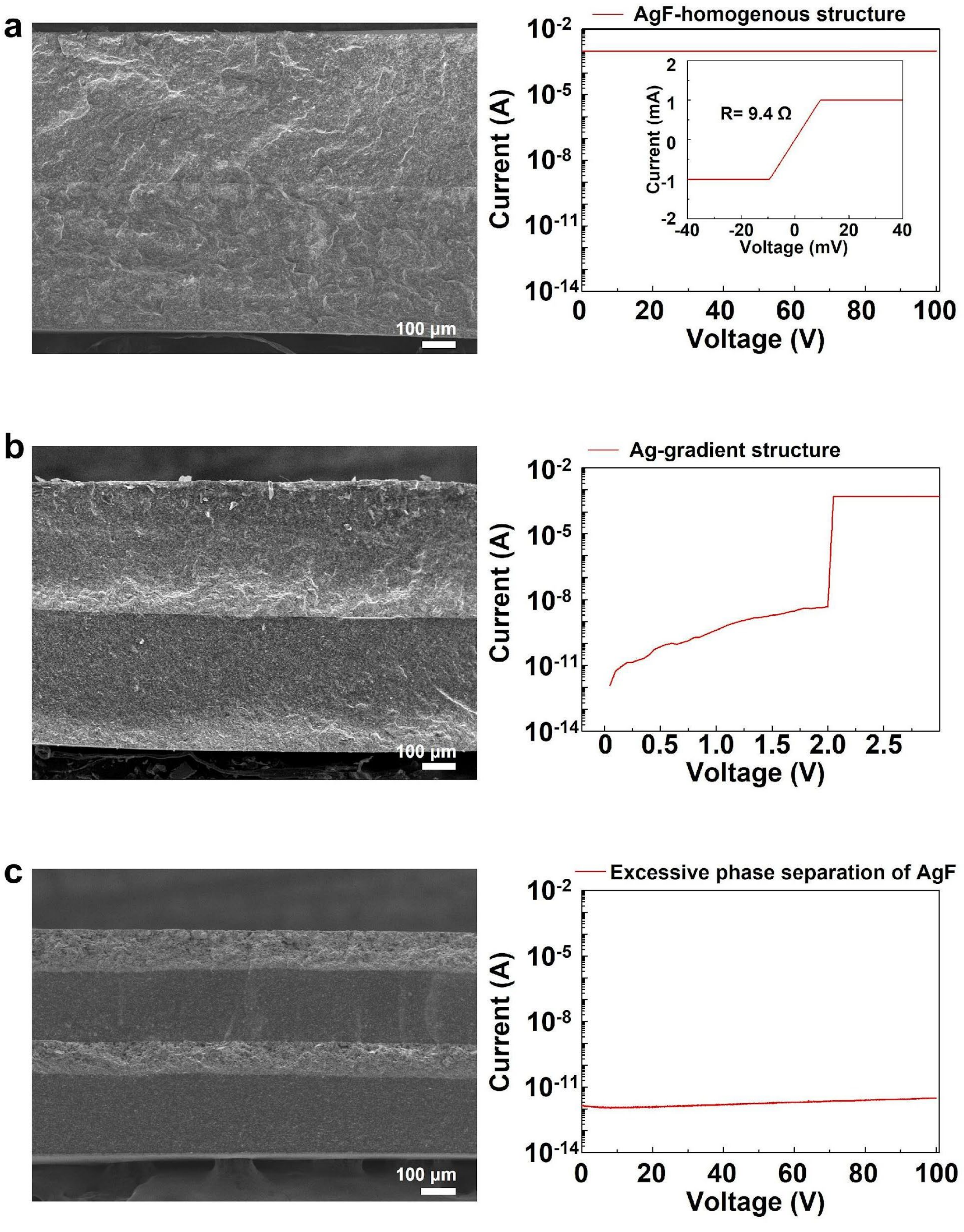

Supplementary Fig. S9 | SEM images and the corresponding I-V curves of the three SS-RRAM devices comprising. a, AgF-homogenous, **b,** AgF-gradient, and **c,** excessively separated AgF layers.

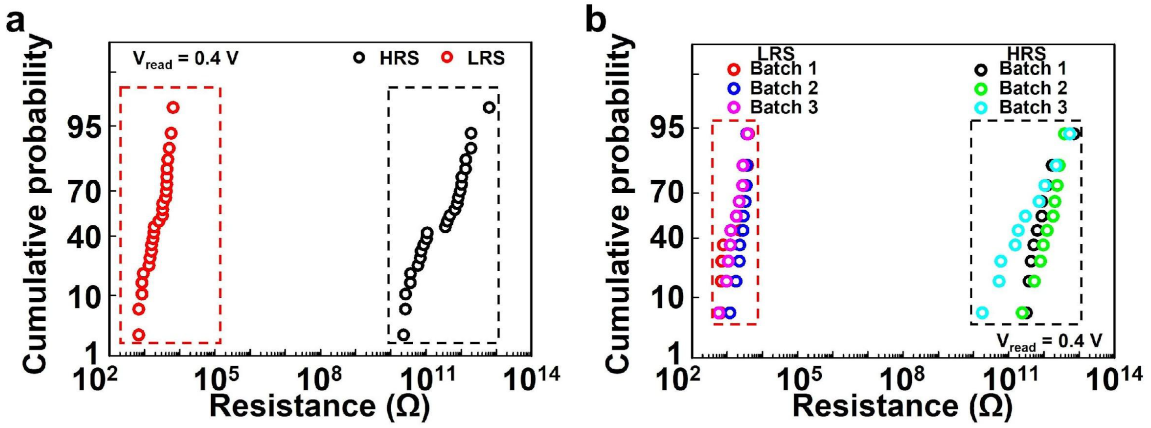

Supplementary Fig. S10 | Areal and batch-to-batch uniformity of fabricated SS-RRAM. a, Cumulative probability of SS-RRAM devices for areal. **b,** Cumulative distribution of SS-RRAM devices for batch-to-batch uniformity.

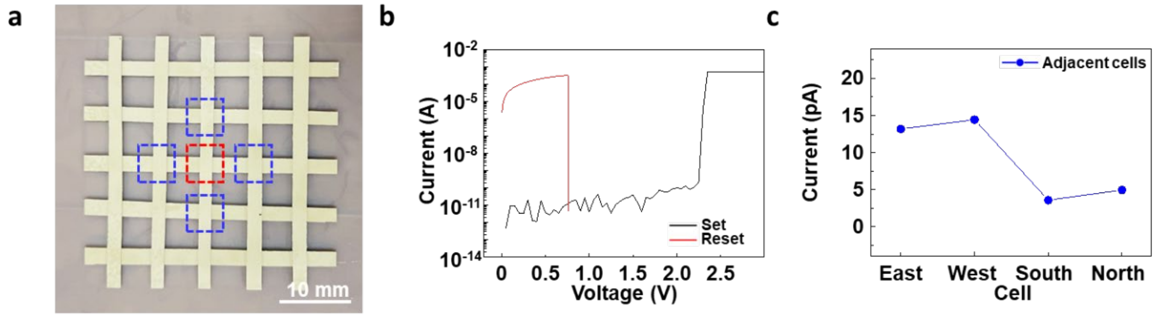

Supplementary Fig. S11 | Characterization of selected and adjacent cells in the 5 × 5 SS-RRAM array. a, Photographic image of 5 × 5 SS-RRAM array. **b,** I-V characteristics of the selected RRAM cell (red dashed box). Blue dashed boxes indicating the neighboring RRAM cells which are set as HRS. **c,** The neighboring RRAM cells showing low current values.

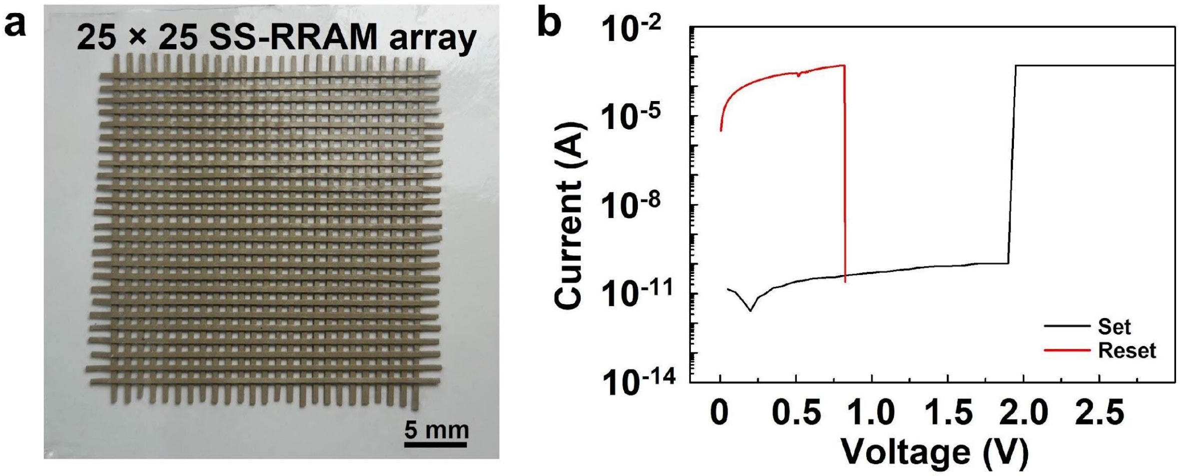

Supplementary Fig. S12 | Fabrication of 25 × 25 SS-RRAM array. a, Image of the 25 × 25 SS-RRAM array. **b,** I-V curve of the selected SS-RRAM cell.

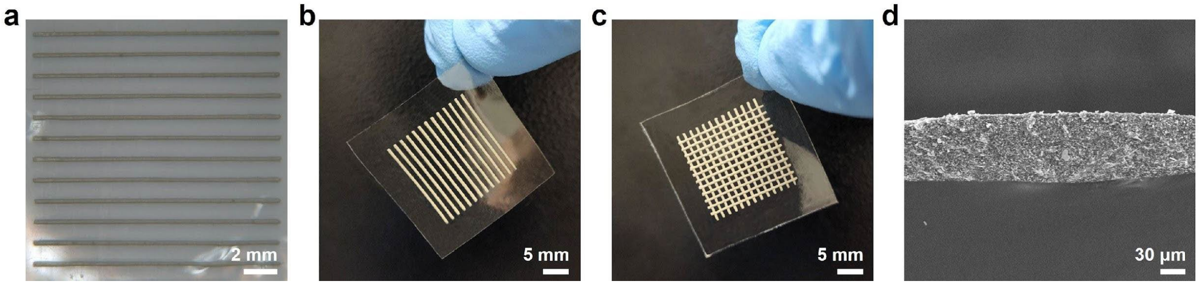

Supplementary Fig. S13 | SS-RRAM array made by screen-printing process. a, Optical and **b,** photographical images of the screen-printed AgF-SHP electrodes, **c,** image of the RRAM array, **d,** and cross-sectional SEM image of the AgF-SHP electrode.

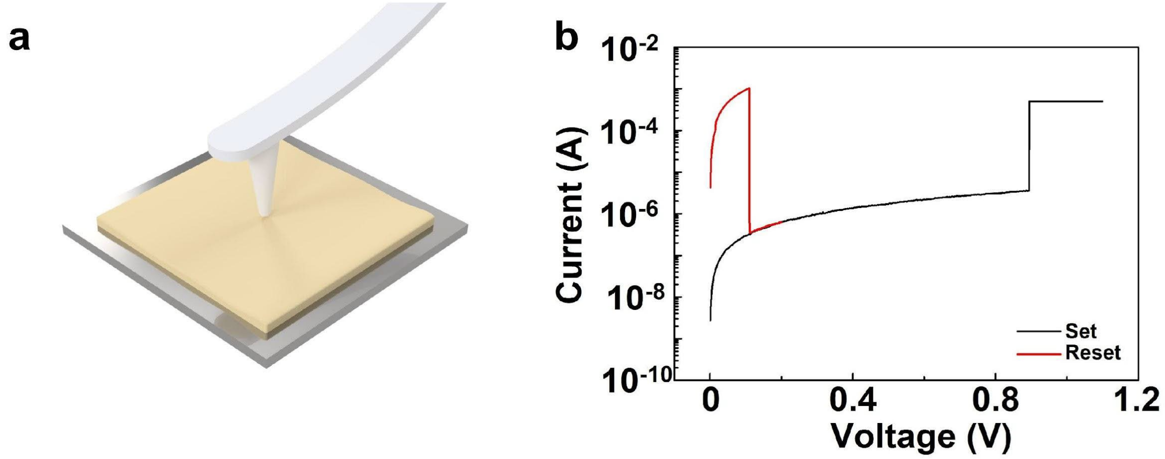

Supplementary Fig. S14 | Schematic illustration and current-voltage characteristic of SS-RRAM in nanoscale. a, Schematic of nanoscale resistive switching using Ag-coated AFM tip. **b,** I-V characteristics of nanoscale RRAM device.

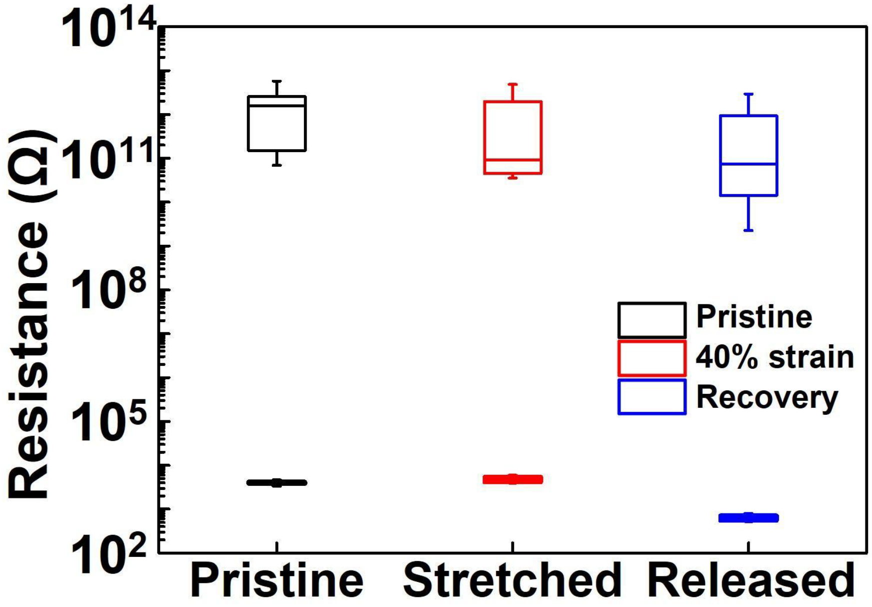

Supplementary Fig. S15 | Electrical characteristic of SS-RRAM under the stretching condition. Dependence of resistance state in sequence of stretching process. The black, red and blue box correspond to HRS and LRS state of the pristine, under 40% strain and after recovered condition, respectively.

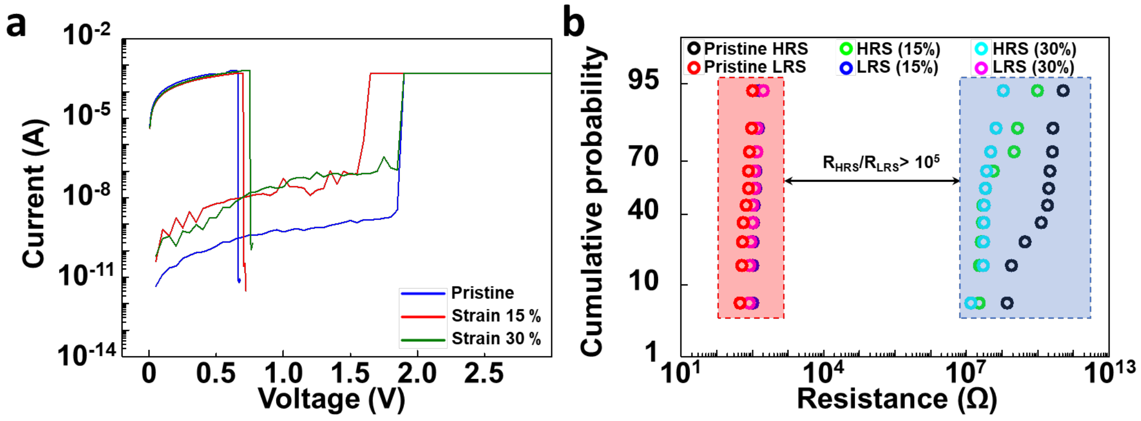

Supplementary Fig. S16 | Electrical characteristics of SS-RRAM under cell-deformed condition without stretching interconnects. a, I-V characteristics of stretched SS-RRAM cells with tensile strain of 0% (blue), 15% (red) and 30% (olive), respectively. **b,** Cumulative probability of LRS and HRS with different cell-stretched conditions.

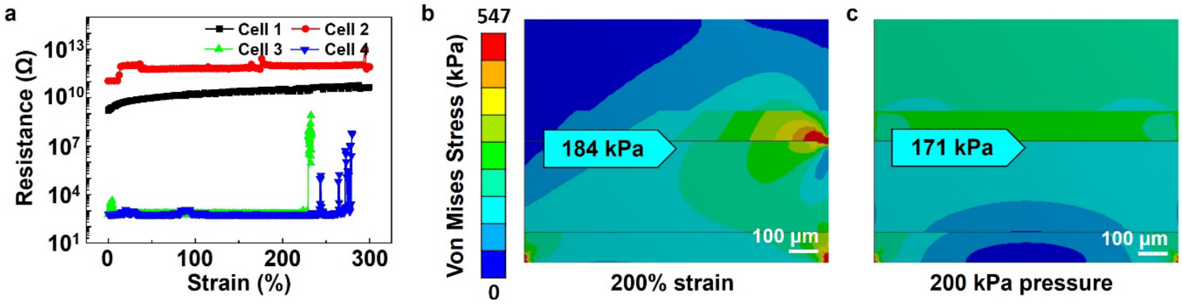

Supplementary Fig. S17 | Experimental results and Simulation of HRS and LRS stability with external mechanical stimuli. a, Plot of resistance of four RRAM cells as a function of strain up to ~300%. **b,** A finite element method (FEM) Simulation of stretched SS-RRAM with a strain of 200%. **c,** A FEM Simulation of SS-RRAM loaded to 200 kPa pressure.

Supplementary Note #2

An electromechanical stability of RRAM device was analytically investigated through a finite element method (FEM) using engineering simulation software (ANSYS, Ansys Inc., Canonsburg, PA, USA) under stretching to 200% strain and compressing to 200 kPa. The FEM results showed that the stress distribution within the switching layer tended to be similar and the electrodynamically stable contour was manifested due to the strain dissipation properties of the self-healing polymer with dynamic hydrogen bond[22] (Supplementary Fig. S17b and S17c). Despite some stress accumulation (under ~184 kPa stress) on the switching layer, the excellent electrical and mechanical stability of the RRAM device could stabilize the operation, matched well with our results in the individual organic RRAM under stretching and compression (Supplementary Fig. S17 and Fig. 3h). These results fully support our materials strategy on intrinsically stretchable RRAM for realizing the strain-insensitive stable memory.

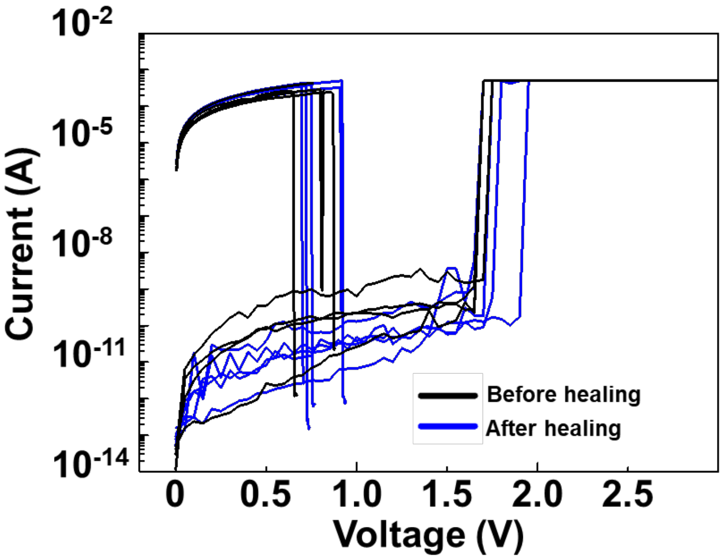

Supplementary Fig. S18 | Current-voltage switching behavior of SS-RRAM before/after self-healing process. The black-colored and blue-colored curves correspond to I-V data before and after the healing process, respectively.

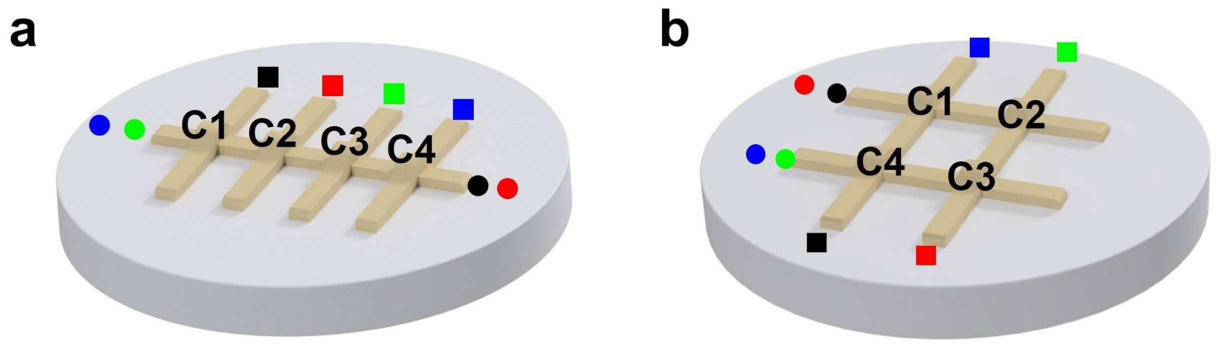

Supplementary Fig. S19 | Schematic illustration of 1 × 4 and 2 × 2 SS-memory array. The colored-rectangles and colored-circles correspond to word-lines and bit-lines in **a,** 1 × 4 and **b,** 2 × 2 SS-RRAM arrays. The black, red, green and blue circles were selected where measuring I-V for Cell 1, Cell2, Cell 3 and Cell 4 in 1 × 4 and 2 × 2 SS-RRAM arrays, respectively.

Supplementary Note #3

To confirm the electrical property of the memory array, we performed the I-V switching test of the memory array before and after conversion. In the resistivity switching test of 1 × 4 array, the farthest end of the word line was probed from the bit line of any testing cell. So that the electrical test possibly included the interference of non-selected memory cells. Each I-V curve of 1 × 4 array was shown in Fig. 4h, in which the electrical behavior was likely to be the same with the 1 × 1 memory cell in Fig. 2b, this result indicated that the resistivity-switching of cells in the 1 × 4 array was also operating well. Next, the measured 1 × 4 array was converted into a 2 × 2 array. After the healing process, I-V switching test of each cell was performed in a rearranged 2 × 2 array as shown in Fig. 4i, Testing lines of each cell were intentionally selected to pass through the healed bit line, so that it confirmed the full operation of 2 × 2 array. The resistivity-switching for each cell remained in the same range of the Set and Reset bias of 1 × 1 memory in Fig. 4b. Figure 4j presented the resistance state of cells in 2 × 2 and 1 × 4 arrays. The resistance of each test was measured with reading one-fifth of set and reset bias. Even

though the resistance of HRS and LRS had a certain distribution for each cell, all memory cells still exhibited the resistance window higher than $\sim 10^5$.

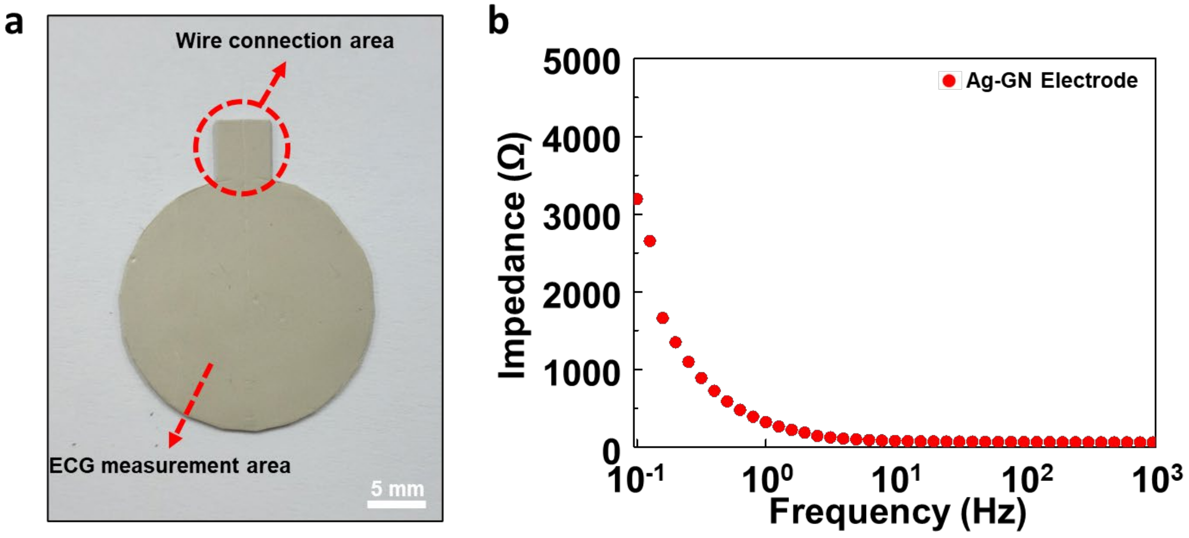

Supplementary Fig. S20 | Ag-GN as an electrocardiogram electrode. a, A photograph of ECG electrode. **b,** Impedance of ECG electrode made by Ag-GN.

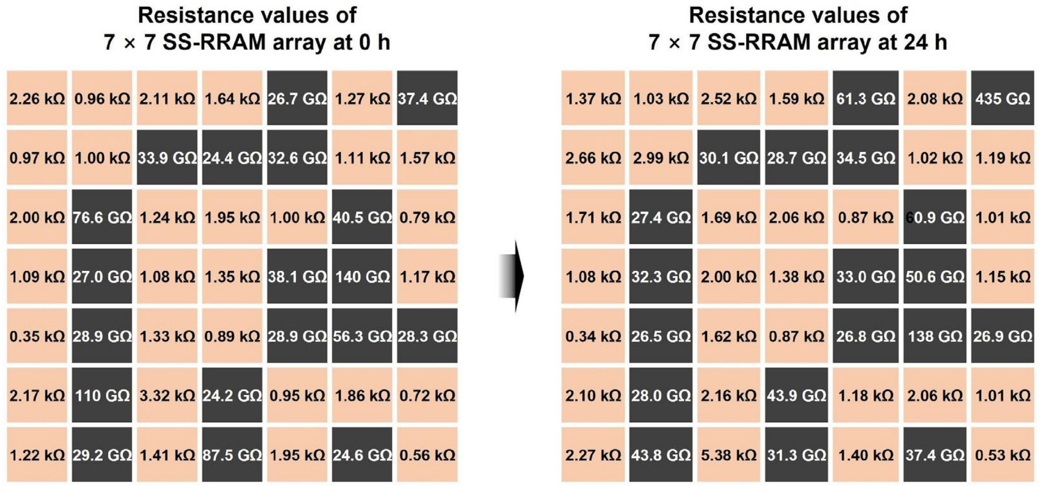

Supplementary Fig. S21 | Binary BPM raw electrical resistance information of 7 × 7 SS-RRAM array. Resistance values of 7 × 7 SS-RRAM array at 0 h (left) and 24 h (right).

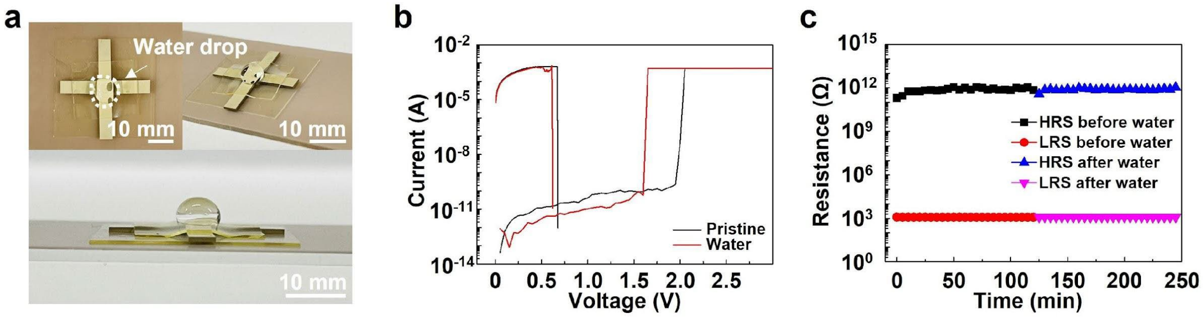

Supplementary Fig. S22 | Waterproof demonstration of encapsulated SS-RRAM. a, Images of the SS-RRAM encapsulated with self-healing polymer film for waterproof demonstration. **b,** The corresponding I-V curve. **c,** Retention data of the SS-RRAM before and after applying a water drop.

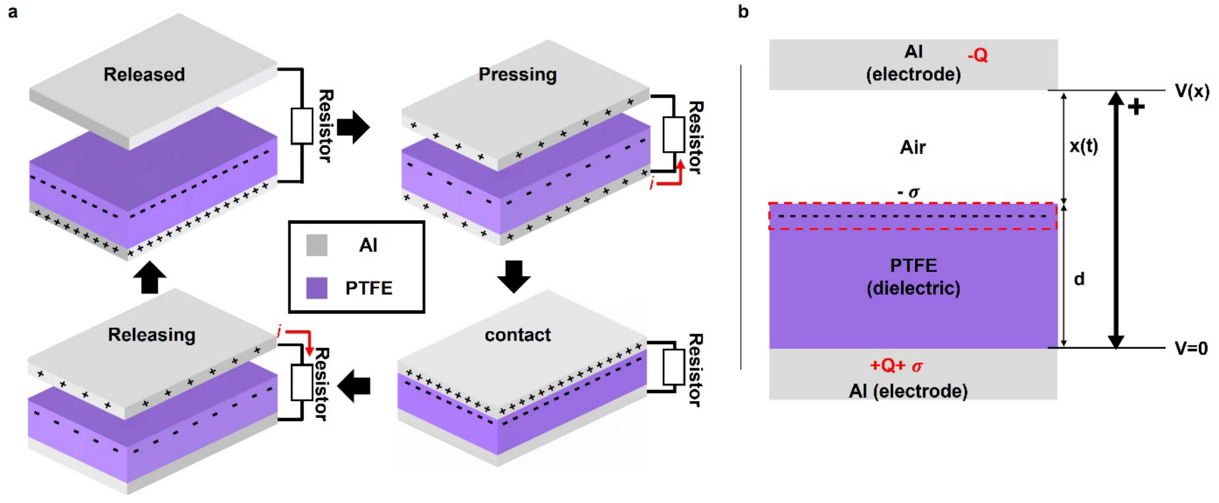

Supplementary Fig. S23 | Working principle and V-Q-X relation of metal-to-dielectric contact mode TENG. a, Schematic representation of the triboelectric generation process, which were the following steps: pressing, contact, releasing and released process. **b,** A simple model for a metal-to-dielectric contact system.

Supplementary Note #4

Working principle

By contacting the metal electrode (Al) and dielectric (PTFE), electrons were transferred from the Al to the PTFE surface. Repetitive contact between the two surfaces induces negative and positive triboelectric charges on the PTFE and Al surfaces, respectively.

Then, the current was promptly generated by the potential difference between the electrodes while the external force was released. Although the external force was fully released, the negative charges induced by triboelectrification remained under ambient conditions.

V (voltage)-*Q* (charge)-*x* (distance) model

As a simple model, the V (voltage)-Q (charge)-x (distance) relationship can be used to interpret the dependence between the output and contact height in a TENG system. The size of the area covered by the metal was assumed to be larger than their separation distance, and the induced tribo-charges were uniformly distributed at the surface with insignificant decay. With the above assumption, the electric field equation can be represented using the parallel plate model and Gaussian theorem. In the conductor-to-dielectric system, the electrostatic field between the top and bottom electrodes was expressed in two parts, namely E_{air} (air gap) and $E_{dielectric}$ (dielectric), given by:

(1) air gap: $E_{air} = -\frac{Q}{S\varepsilon_0} + \frac{\sigma}{\varepsilon_0}$

(2) dielectric: $E_{dielectric} = -\frac{Q}{S\varepsilon_0\varepsilon_d}$

where Q, S, ϵ_0, $\sigma(t)$, and ε_d are the amount of charge transferred between two electrodes, the size of the metal, permittivity of air, surface tribo-charge density, and permittivity of the dielectric, respectively. The potential difference between the two electrodes can be expressed as follows:

(3) $V = E_{dielectric}d + E_{air}x(t) = -\frac{Q}{S\varepsilon_0}\left(\frac{d}{\varepsilon_d} + x(t)\right) + \frac{\sigma x(t)}{\varepsilon_0}$

where d and $x(t)$ are the thickness of the dielectric film and the distance of the air gap, respectively.

In the case of open-circuit measurements, the charge transferred between the two electrodes is zero (Q=0). Thus, the open-circuit voltage (V_{OC}) can be written as follows:

(4) $V_{OC} = \frac{\sigma x(t)}{\varepsilon_0}$

In the application of self-powered SS-RRAM triggering, we controlled the distance x to adjust the TENG power output.

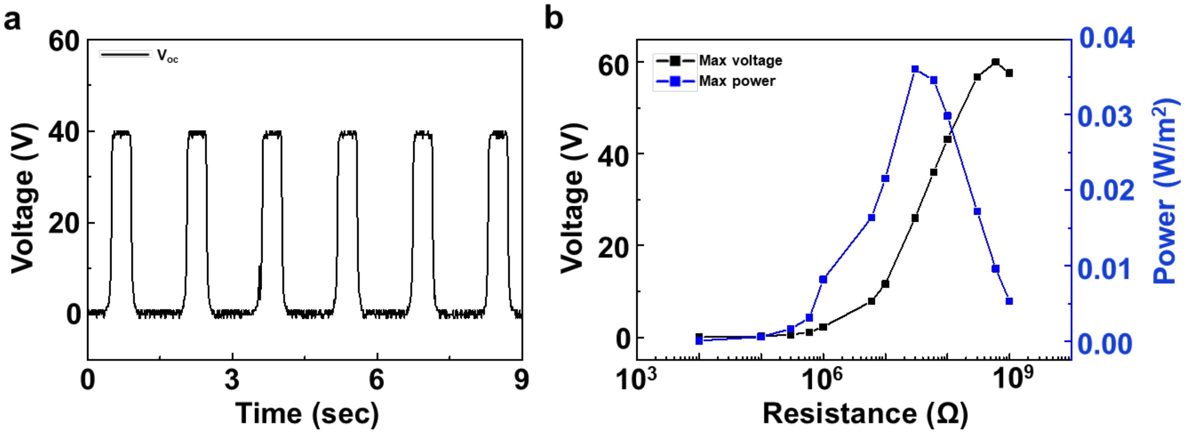

Supplementary Fig. S24 | Energy harvest characteristic of TENG based PTFE and Al.

a, Open circuit voltage of TENG. **b,** Output voltage and power density of TENG on the different road resistance values.

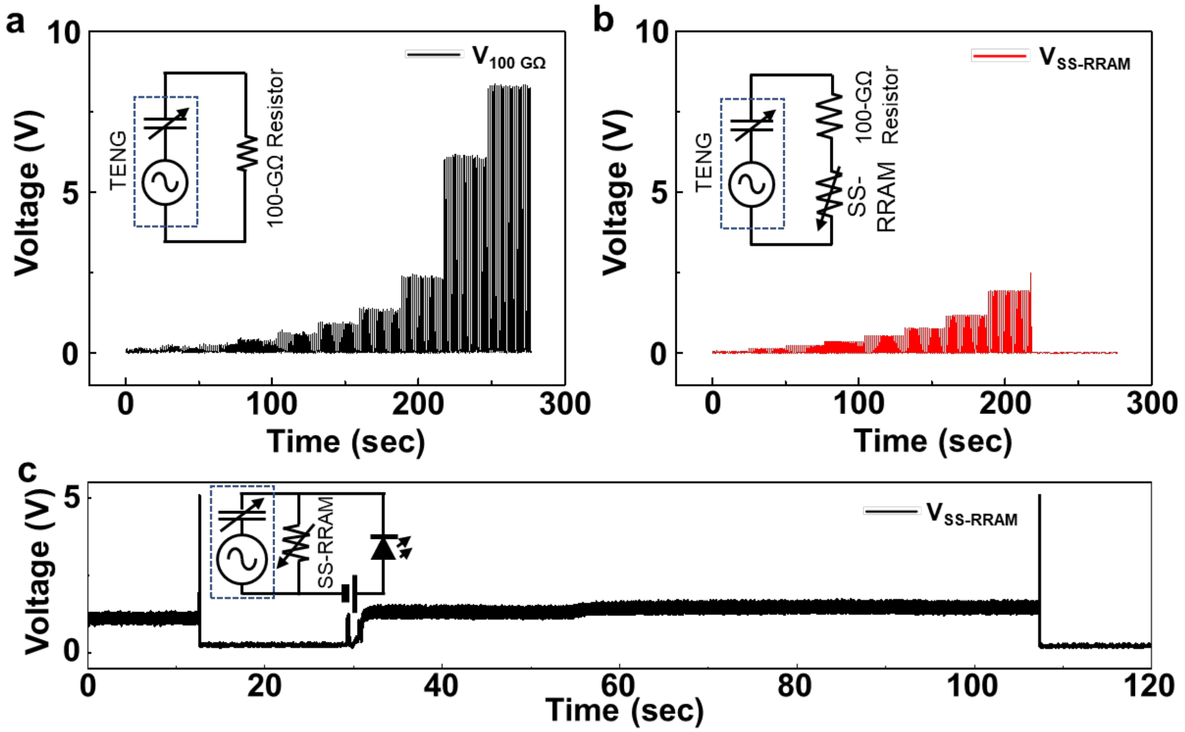

Supplementary Fig. S25 | Voltage drops at SS-RRAM switched from HRS to LRS. a, Voltage drop at a 100-GΩ resistor depends on mechanical moving distance of a TENG. **b,** Voltage drop at a SS-RRAM depends on mechanical moving distance of a TENG. When enough power was delivered to the RRAM HRS switched to LRS. Thus, only a minor voltage drop was observed after the switching. **c,** *In-situ* voltage drop at the SS-RRAM in a self-powered system.

Supplementary Note #5.

Prior to the integration of all components, a TENG was tested depending on pushing height at 100 GΩ and thus proper condition of power outputs could be selected for triggering RRAM. The output voltage of TENG increased as the deviation of pushing height increased. Each height was tested 20 cycles. Afterward the RRAM in HRS was connected to a circuit, the triggering voltage was found to be ~2 V, which was mostly identical value with the former I-V test shown in Figure 2g. Once the HRS of RRAM changes to LRS during the operation of TENG voltage crossed

RRAM should be dropped instantaneously. Because the resistance of LRS was much lower than a 100-GΩ resistor in the circuit.

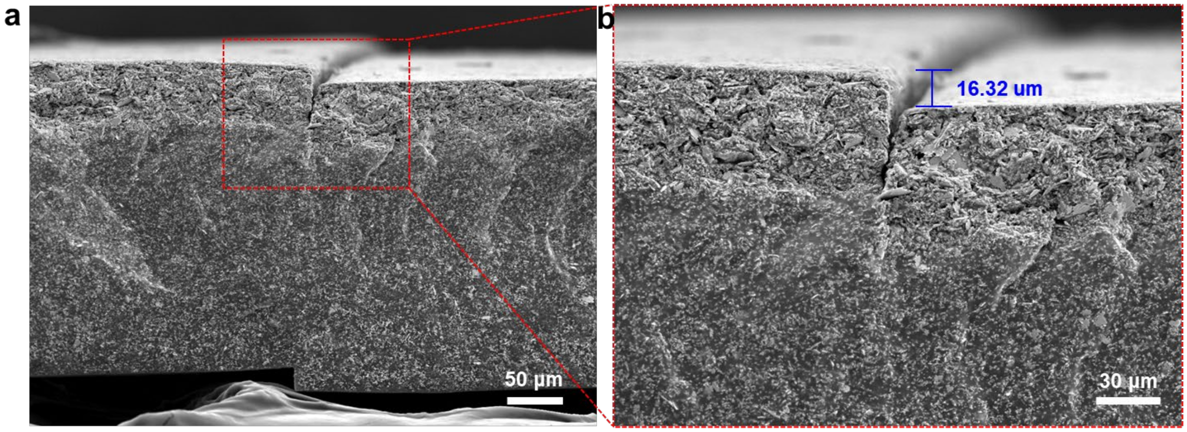

Supplementary Fig. S26 | SEM image of AgF-SHP composite electrode. a, Cross-sectional SEM image of reconnected Ag-GN. **b,** The magnified SEM image of reconnected Ag-GN.

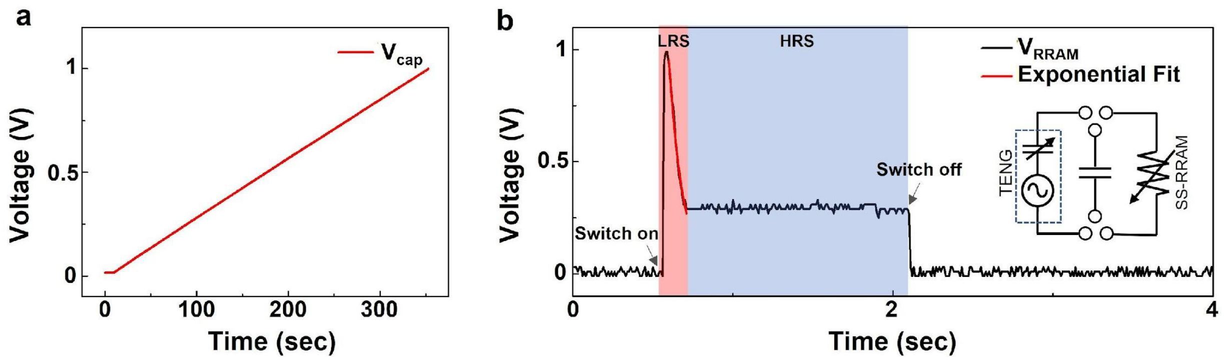

Supplementary Fig. S27 | Charging and discharging characteristic of integrated SS-RRAM with TENG and capacitor. a, A Charging curve of a 100-μF capacitor using a TENG. **b,** A *voltage-time* curve represents a 'Reset' process of a SS-RRAM. A circuit was composed of a TENG, capacitor, and SS-RRAM.

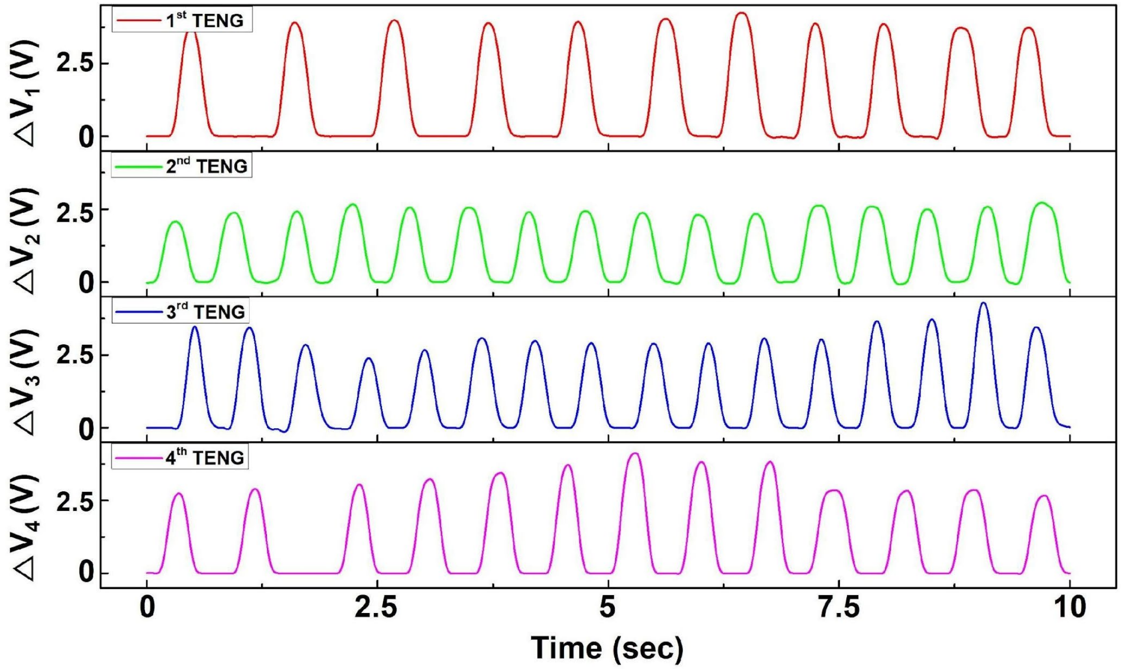

Supplementary Fig. S28 | The voltage outputs generated by finger tapping on TENGs. The evoked sensing signals in the individual voltage output results (red, 1st TENG; green, 2nd TENG; blue, 3rd TENG; pink, 4th TENG) were clearly verified under finger tapping-induced stimuli.
